# Clinical implications of 10-formyltetrahydrofolate dehydrogenase expression in hormone receptor-positive breast cancer

**DOI:** 10.3389/fonc.2026.1838093

**Published:** 2026-05-25

**Authors:** Han Suk Ryu, Amira A. Abdellatef, Da Sol Kim, Ilias P. Nikas, Sergey A. Krupenko

**Affiliations:** 1 Nutrition Research Institute, University of North Carolina at Chapel Hill, Kannapolis, NC, United States; 2 Department of Pathology, Seoul National University Hospital, Seoul National University College of Medicine, Seoul, Republic of Korea; 3 Medical School, University of Cyprus, Nicosia, Cyprus; 4 Department of Nutrition, University of North Carolina at Chapel Hill, Chapel Hill, NC, United States

**Keywords:** ALDH1L1, biomarker, hormone receptor-breast cancer, prognosis, tumor suppressor gene

## Abstract

**Objective:**

ALDH1L1, a key enzyme in folate metabolism, has been implicated in various cancers, but the clinical significance of its expression in breast cancer remains unclear.

**Methods:**

We analyzed three cohorts from Seoul National University Hospital: (i) 41 non-matched patient samples (23 samples from normal mammary tissues and 18 samples from invasive carcinoma); (ii) 44 paired normal and invasive mammary carcinoma patient tissues; (iii) tissue microarray of 1,001 invasive ductal carcinoma patients. ALDH1L1 immunostaining was performed on 1,086 cases (combined samples from the three cohorts) using tissue microarrays or whole section slides with positive cytoplasmic and membranous reactivity. Clinical (tumor size, grade, lymphatic invasion, and lymph node metastasis) data were collected from patient records.

**Results:**

ALDH1L1 expression was higher in non-tumor tissues than cancer tissues (p=0.0014 and p=0.0282 for two datasets), and inversely correlated with increased tumor size, advanced stage, and lymphatic spread. Higher ALDH1L1 expression is associated with smaller tumor size, lower pT stage in luminal A and HER2+ subtypes, and lower nuclear grade in triple-negative breast cancer. ALDH1L1 expression was associated with improved overall and disease-free survival, particularly in hormone receptor-positive subtypes (p=0.0049 and p=0.0441). These findings were confirmed by METABRIC database analysis. In agreement with the association between ALDH1L1 expression and tumor aggressiveness, proliferation/clonogenic assays showed strong cytotoxic effects of lentivirus-delivered ALDH1L1 in MCF7 and T47D luminal breast cancer cells.

**Conclusion:**

Reduced ALDH1L1 expression is associated with aggressive clinicopathologic features and poorer survival in breast cancer, with a particularly evident and clinically relevant prognostic impact in the luminal A subtype. These findings highlight ALDH1L1 as a subtype-specific favorable biomarker and a potential therapeutic target in hormone receptor–positive breast cancer.

## 1 Introduction

Though recent progress in targeted therapy and novel biomarkers for clinical trials has provided promising therapeutic strategies for breast cancer treatment (1), breast cancer remains the most commonly diagnosed malignancy and the second most fatal cancer among women worldwide (2). Common targeted therapies for breast cancer nowadays include inhibitors of epidermal growth factor receptor (EGFR) family members, hormone receptor ligands, and cell cycle regulators (reviewed in (3–7)). In addition, immunotherapy, including immune checkpoint inhibitors, as well as antibody-drug conjugates (ADCs), and emerging cellular therapies, has shown clinical promise across breast cancer subtypes, with ongoing trials expanding their use in hormone receptor-positive, HER2-positive, and triple-negative breast cancers (8, 9). Recent evidence also suggests that restoring inactivated tumor suppressor genes may represent a novel therapeutic strategy for combating mammary tumor cells (10).

Breast cancer is a heterogeneous disease that comprises several intrinsic molecular subtypes with distinct biological and clinical characteristics. Based on hormone receptor and HER2 (Human Epidermal Growth Factor Receptor 2) expression, tumors are broadly classified into luminal A, luminal B, HER2-positive, and triple-negative breast cancer (TNBC) (11). These subtypes differ in proliferation rate, aggressiveness, prognosis, and therapeutic strategies. Luminal tumors generally exhibit hormone receptor positivity and are treated primarily with endocrine therapy, whereas HER2-positive and TNBC subtypes tend to demonstrate more aggressive behavior and often require systemic chemotherapy and/or targeted therapy. Given these differences, identifying subtype-specific biomarkers is essential for improving risk stratification and therapeutic decision-making (Reviewed in (11, 12)). The key clinicopathological characteristics of these intrinsic subtypes are summarized in **Table 1**. This molecular and clinical heterogeneity underscores the importance of identifying biomarkers that may have subtype-specific prognostic or therapeutic relevance.

**Table 1:** Clinicopathological characteristics of intrinsic breast cancer subtypes.

Variables	Luminal A	Luminal B	HER2+	TNBC
ER	+	+	–	–
PR	+	+/-	–	–
HER2	–	+/-	+	–
Proliferation	Low	Intermediate	High	High
Aggressiveness	Low	Intermediate	High	Very high
Prognosis	Favorable	Moderate	Historically poor (improved with anti-HER2 therapy)	Poor

*ALDH1L1* (aldehyde dehydrogenase 1 family, member L1) has emerged as a candidate tumor suppressor whose expression is frequently suppressed in multiple malignancies. Epigenetic silencing of ALDH1L1 through promoter CpG island hypermethylation has been reported in hepatocellular carcinoma, lung cancer, glioblastoma, and breast cancer, and is associated with reduced mRNA and protein expression levels (13–18). In several tumor models, treatment with DNA demethylating agents partially restored ALDH1L1 expression, supporting promoter methylation as a major regulatory mechanism (14, 16). In addition to transcriptional repression, ALDH1L1 protein stability is regulated in a cycle–dependent manner through ubiquitin-mediated proteasomal degradation, indicating multilayer control of its tumor-suppressive function (19). This gene encodes 10-formyltetrahydrofolate dehydrogenase, a key enzyme involved in folate metabolism (20). ALDH1L1 catalyzes the NADP^+^-dependent conversion of 10-formyltetrahydrofolate to tetrahydrofolate, generating NADPH and carbon dioxide, thereby regulating the availability of one-carbon units for biosynthetic processes (19, 20). In the cells, folate coenzymes play a pivotal role in numerous one-carbon transfer reactions, contributing to the biosynthesis of amino acids and nucleotides. Folate is also required for mitochondrial translation (21) and contributes to NADPH production (22). Importantly, the folate-dependent regeneration of methionine from homocysteine, required for the biosynthesis of the universal methyl donor S-adenosylmethionine (SAM), links folate metabolism to cellular methylation processes (23–25). As higher animals cannot synthesize folate, they rely on dietary intake of this essential vitamin.

Folate-dependent pathways, including methylation and nucleotide biosynthesis, are critical for rapidly dividing cells; therefore, cancer cells are highly susceptible to folate availability and disruption of folate metabolism (26). Malignant tumors frequently upregulate folate pathways by overexpressing certain folate-related enzymes to meet the increased demand for folate-dependent one-carbon transfer reactions (25, 27). ALDH1L1, however, competes with folate-dependent biosynthesis, thus restricting cellular proliferation (reviewed in (19, 28)). Therefore, the downregulation of this enzyme in malignant tumors is likely to provide an advantage to cancer cells over normal cells (19). Although previous studies have reported altered ALDH1L1 mRNA in breast cancer (18, 29, 30), the clinical and subtype-specific significance of the protein expression remains incompletely understood. The present study aimed to assess the clinical relevance of ALDH1L1 in large cohorts of patients with breast cancer and to validate its prognostic role in this cancer type by analyzing publicly available datasets.

## 2 Materials and methods

### 2.1 Cancer patient cohorts and study design

Three independent patient cohorts from Seoul National University Hospital were included in this study, with no overlapping cases among the cohorts. The first cohort included normal mammary tissue samples (NMT) from 23 patients and invasive mammary carcinoma samples (IMC) from 18 patients. The second cohort included 44 paired normal and tumor tissue samples from patients with invasive mammary carcinoma. These two cohorts were used to compare ALDH1L1 expression in non-tumorous mammary gland tissue and carcinoma cells. The third cohort was the breast cancer tissue microarray, consisting of 1,001 patients diagnosed with invasive ductal carcinoma, who underwent surgical resection between 2009 and 2012. This analysis aimed to assess the correlation between ALDH1L1 expression and clinicopathological parameters in breast cancer. For histological and immunohistochemical analyses, tissue microarrays consisting of 2 mm cores from the cases above were constructed (Superbiochips Laboratories, Seoul, Korea). The electronic medical records system retrieved clinicopathological parameters and patient survival data, including nuclear grade, histological grade, tumor size, T stage, lymphovascular tumor emboli, and lymph node metastasis. Nuclear and histological grading were based on the Nottingham grading system (31). Cases were classified according to the 8^th^ edition of the American Joint Committee on Cancer (AJCC) (32). Patients who had received neoadjuvant chemotherapy or radiotherapy were excluded from the analysis. All enrolled patients had previously been subjected to estrogen receptor (ER) and progesterone receptor (PR) immunohistochemical analysis, as well as human epidermal growth factor receptor 2 (HER2) immunohistochemical and/or *in situ* hybridization testing. Results were interpreted based on the 13^th^ St. Galen International Breast Cancer Conference and ASCO/CAP guidelines (33). Briefly, ER (1:100, 1D5; Novocastra Laboratories, Newcastle, UK) and PR (1:200, PgR636; Dako, Glostrup, Denmark) expressions were recorded as positive when ≥ 1% of cancer cells showed nuclear staining of any intensity (34). HER2 immunohistochemistry (1:1, 4B5; Ventana, Medical System, Tucson, AZ, USA) was regarded as positive (3+) when ≥10% of the tumor cells showed complete and intense circumferential membranous staining based on the 2018 ASCO/CAP guidelines (33). Equivocal cases (2+) were further evaluated by confirmatory fluorescence *in situ* hybridization (FISH) using the PathVysion assay (Abbott Molecular, Downers Grove, IL, USA). According to ASCO/CAP guidelines, a HER2 test is classified as positive when the HER2/CEP17 ratio is ≥ 2.0 or the average HER2 gene copy number is ≥ 6.0 (33).

### 2.2 Immunohistochemistry staining

Immunohistochemical staining was performed on 1,001 samples arranged in tissue microarrays consisting of 2 mm cores from the aforementioned cases (Superbiochips Laboratories, Seoul, Korea) or 85 whole-section slides. Subsequently, 4-μm sections from each block were subjected to immunohistochemistry (IHC) using in-house ALDH1L1-specific polyclonal antibody (1:350) (13, 35) and a Benchmark automatic immunostaining device (Ventana, Arizona, USA). Cytoplasmic and membranous staining was evaluated and scored based on both the intensity and the percentage of positive tumor cells using the H-score method, as described below. Immunoreactivity scores were determined by multiplying the percentage of immunoreactive tumor cells by the intensity of immunoreactivity (0, negative; 1, weakly positive; 2, moderately positive; 3, strongly positive). The immunohistochemistry (IHC) results were calculated for each case using the following formula, ranging from 0 to 300: H-Score = (IS_0 × PS_0) + (IS_1 × PS_1) + (IS_2 × PS_2) + (IS_3 × PS_3) (36). The cut-off for low and high ALDH1L1 expression was set at an H-score of 150. To enhance accuracy, all immunohistochemical staining results were interpreted independently by experienced breast pathologists (H.S.R. and I.P.N.). Any discordance between the two was resolved by a consensus.

### 2.3 Public database

Publicly available datasets for human patients with breast cancer and breast cancer cell lines, including METABRIC and the Cancer Cell Line Encyclopedia (CCLE), were accessed and analyzed using cBioPortal (http://www.cbioportal.org/) and CCLE (https://sites.broadinstitute.org/ccle/). Disease-free survival (DFS) and overall survival (OS) of breast cancer patients in the METABRIC datasets were analyzed using Cancer Target Gene Screening (CTGS; http://ctgs.biohackers.net). Folate‐metabolizing enzymes and transporters in breast cancer and normal tissues, as well as the correlation analysis between ALDH1L1 expression and a set of breast cancer marker proteins, were evaluated using the GEPIA2 platform (http://gepia2.cancer-pku.cn/#index). Data on mRNA expression levels of folate-metabolizing enzymes in luminal breast cancer cells and ALDH1L1 mRNA expression in selected breast cancer cells were retrieved from the Human Protein Atlas database (https://www.proteinatlas.org). The list of cancer-associated genes was obtained from the OncoKB™ Cancer Gene List platform (https://www.oncokb.org/). Gene Ontology enrichment analysis was performed using the publicly available STRING database (https://string-db.org/).

### 2.4 Cell lines and cultures

Human breast cancer cell lines MCF7, T47D, SKBR3, MDA-MB-231, and MDA-MB-468, as well as HEK-293a cells, used in this study, were from the American Type Culture Collection (ATCC) and were cultured for no more than 8–10 sequential passages. All breast cancer cell lines were cultured in F12K Nutrient Mixture Kaighn’s medium (Thermo Fisher Scientific, Waltham, MA, USA). HEK-293a cells were cultured in Dulbecco’s Modified Eagle’s Medium (DMEM). All media were supplemented with 10% fetal bovine serum (Bio-Techne, Minneapolis, MN, USA) and a 1% antibiotic-antimycotic solution (Thermo Fisher Scientific). Cell cultures were maintained at 37 °C in a humidified atmosphere with 5% CO_2_. All cell lines used in this study were mycoplasma-free.

### 2.5 Plasmid constructions and amplification

Custom lentiviral vector carrying human ALDH1L1 cDNA was purchased from Genecopia (Rockville, MD, #EX-Q0477-Lv102-B). In this construct, the ALDH1L1 ORF (NM_012190.3/XM_024453325.2) was inserted into the pReceiver-Lv201 vector (Genecopia, #EX-Q0477-Lv102-B) downstream of the CMV promoter. The resulting vector contained eGFP under the control of SV40 promoter, ALDH1L1 under the control of CMV promoter, and the puromycin resistance elements for selection (37).

### 2.6 Transfection and lentivirus packing

Recombinant lentivirus was packaged in HEK-293a cells using the Lenti-Pac™ HIV Expression Packaging Kit (Genecopoeia, LT002) according to the manufacturer’s protocol as described previously (37). Briefly, 2.5 μg of lentiviral vector was diluted in 200 μL of Opti-MEM^®^ I (Gibco, Waltham, MA, USA, #A36351-01) and mixed with 5 μL (0.5 μg/μL) of Lenti-Pac HIV in a sterile polypropylene tube. In a separate tube, 15 μL of EndoFectin Lenti was diluted in 200 μL of Opti-MEM I. The diluted EndoFectin Lenti reagent was then added dropwise to the DNA solution while gently vortexing the tube. The mixture was incubated for 10–25 min at room temperature to allow the DNA-EndoFectin complex to form. HEK-293a packaging cells grown in a T-75 flask to about 70% confluency were transfected by adding the entire DNA-EndoFectin Lenti complex and incubated overnight (8–14 h). Cells were placed in a fresh medium with 1/500 volume of TiterBoost reagent and incubated for an additional 24 h. Pseudovirus-containing culture medium was collected in sterile capped tubes at 36 and 48 h post-transfection. The medium was centrifuged at 500 x g for 10 min to remove cell debris, and the supernatant was filtered through 0.45 μm polyethersulfone (PES) low protein-binding filters. The virus particles were concentrated using Lenti-Pac™ Lentivirus Concentration Solution (6x) (Genecopoeia, LT007). The titer of the harvested lentiviral stocks was determined by RT-PCR using the Lenti-Pac™ HIV qRT-PCR Titration Kit (Genecopoeia, LT005).

### 2.7 Cell proliferation assay

Cell viability was assessed using a CCK-8 cell proliferation assay (Dojindo Laboratories Co., Ltd.). Cells were plated at a density of 5x10^3^ cells/well in a 96-well plate, and CCK-8 was added at specified time points. The plates were processed according to the manufacturer’s instructions. Absorbance at 570 nm was measured using a Wallace 1420 multilabel counter (PerkinElmer Life Sciences).

### 2.8 Colony formation assay

Cells were seeded in a six-well plate (500–1000 cells per well) and cultured for two weeks. Colonies were fixed with an ice-cold methanol-acetone mixture (1:1) and stained with 0.1% crystal violet (Solarbio). The number of colonies was counted using ImageJ, version 1.54g (38). Data are presented as mean ± SEM of three independent experiments.

### 2.9 Wound healing assay

Cells (2x10^5^ cells/well) were seeded in a 12-well plate and cultured for 24 h to form a monolayer (80-90% confluency). Scratches were made by a sterile pipette tip, followed by washing with PBS to remove detached cells and debris; fresh medium (1 mL) was added to each well. Images were captured at 0 hours and again at 24 and 48 hours to assess the extent of wound healing. The percentage of wound closure was calculated as follows: wound closure (%) = [1 − (wound area at 24 or 48 h/wound area at 0 h)] × 100. Here, 0 h represents the time immediately after wounding, and 24 or 48 h is the time point after incubation. Wound closure was expressed as a percentage of the initial wound area. The wounded area was quantified manually using ImageJ, version 1.54g.

### 2.10 Western blot assay

Cells were lysed in RIPA buffer (Thermo Fisher Scientific) containing protease and phosphatase inhibitor cocktails (Thermo Fisher Scientific). The protein content was measured using a bicinchoninic acid (BCA) assay (Thermo Fisher Scientific). Samples were subjected to SDS-PAGE on an 8–16% precast Criterion gel (Bio-Rad, Hercules, CA, USA), followed by transfer to a PVDF membrane (Millipore, Burlington, MA, USA). The membrane was blocked in TBST buffer with 3% dry milk, incubated overnight with either in-house ALDH1L1-specific antibody (1:8000) or antibodies against p21, p27, PARP, and E-Cadherin (1:1000, Cell Signaling), followed by incubation with horseradish peroxidase-conjugated secondary antibody (Cytiva, Marlborough, MA, USA) for 1 h. Actin-specific antibody (1:20,000; Abcam, AB49900) was used to assess actin levels in all samples. After incubation with chemiluminescent substrate (Millipore), specific bands were visualized using an Odyssey FC Imaging System (LI-COR Biosciences, Lincoln, NE, USA). The protein bands were quantified using Image Studio Lite Software (LI-COR Biosciences).

### 2.11 Statistical analysis

All statistical analyses were performed using GraphPad Prism version 10.3.1 (GraphPad Software, San Diego, CA) and R software (version 4.5.2). Two-group comparisons were performed using an unpaired Student’s t-test (two-tailed). For multiple groups, one-way analysis of variance (ANOVA) with Tukey’s correction for multiple comparisons was applied. For survival analysis, Kaplan-Meier survival curves were generated using GraphPad Prism 10, and the log-rank test was performed to assess statistical significance between groups. To evaluate the independent prognostic significance of ALDH1L1 expression, Cox proportional hazards regression analyses were performed using R. Both univariate and multivariate Cox regression models were applied. Multivariate models were adjusted for established clinicopathological prognostic factors, including age, tumor stage, nodal stage, histologic grade, lymphatic invasion, and Ki-67 index. Hazard ratios (HRs) and 95% confidence intervals (CIs) were calculated. The proportional hazards assumption was evaluated using Schoenfeld residuals. Statistical significance was set at p<0.05. Correlation analyses were performed using Pearson’s or Spearman’s correlation tests, as appropriate. For the METABRIC dataset, multiple testing correction was performed using the Benjamini–Hochberg false discovery rate (FDR) method.

## 3 Results

### 3.1 Patient characteristics

Clinicopathological characteristics of the patients in our largest cohort (1,001 patients) are summarized in **Table 2**. All patients were female, with ages ranging from 25 to 90 years (mean: 51.31 years). The cohort consisted of 548 (59.5%) cases of luminal A, 123 (13.4%) cases of luminal B, 83 (9%) cases of HER2-positive, and 167 (18.1%) cases of triple-negative breast cancer (TNBC). Most cases had a nuclear score of 3 (59.2%) and a histological grade of 3 (55.2%) according to the Nottingham histological grade. A total of 347 (35%) and 337 (33.7%) patients had lymphovascular tumor emboli and lymph node metastasis, respectively. The tumor stage I was diagnosed in 315 (31.5%) patients, stage II in 586 (58.5%) patients, and stage III in 100 (10%) patients. ALDH1L1 expression was detected in 622 patients (92.7%) with hormone receptor (HR)-positive breast cancer, 79 (96.3%) HER2-positive patients, and 153 (92.7%) HR- and HER2-negative patients.

**Table 2:** Clinicopathologic characteristics of breast cancer patients.

Variables	Number (%)
Age (years)	
< 50	483 (48.3%)
≥ 50	517 (51.6%)
Unknown	1 (0.1%)
Tumor size (cm)	
≤ 2	501 (50%)
> 2	500 (50%)
Nuclear score	
Score 1	19 (1.9%)
Score 2	389 (38.9%)
Score 3	593 (59.2%)
Histologic grade	
Grade 1	57 (5.7%)
Grade 2	391 (39.1%)
Grade 3	553 (55.2%)
Lymphovascular tumor emboli	
Absent	645 (64.4%)
Present	347 (34.7%)
Unknown	9 (0.9%)
Lymph node metastasis	
Absent	664 (66.3%)
Present	337 (33.7%)
pT stage	
Stage I	315 (31.5%)
Stage II	586 (58.5%)
Stage III	100 (10.0%)
AJCC stage	
Stage I	387 (38.7%)
Stage II	587 (58.6%)
Stage III	27 (2.7%)
Intrinsic subtype	
Luminal A	548 (54.7%)
Luminal B	123 (12.3%)
HER2+	83 (8.3%)
TNBC	167 (16.7%)
Unknown	80 (8%)

### 3.2 Expression of ALDH1L1 in breast cancer and non-cancerous breast tissues

**Figure 1:**
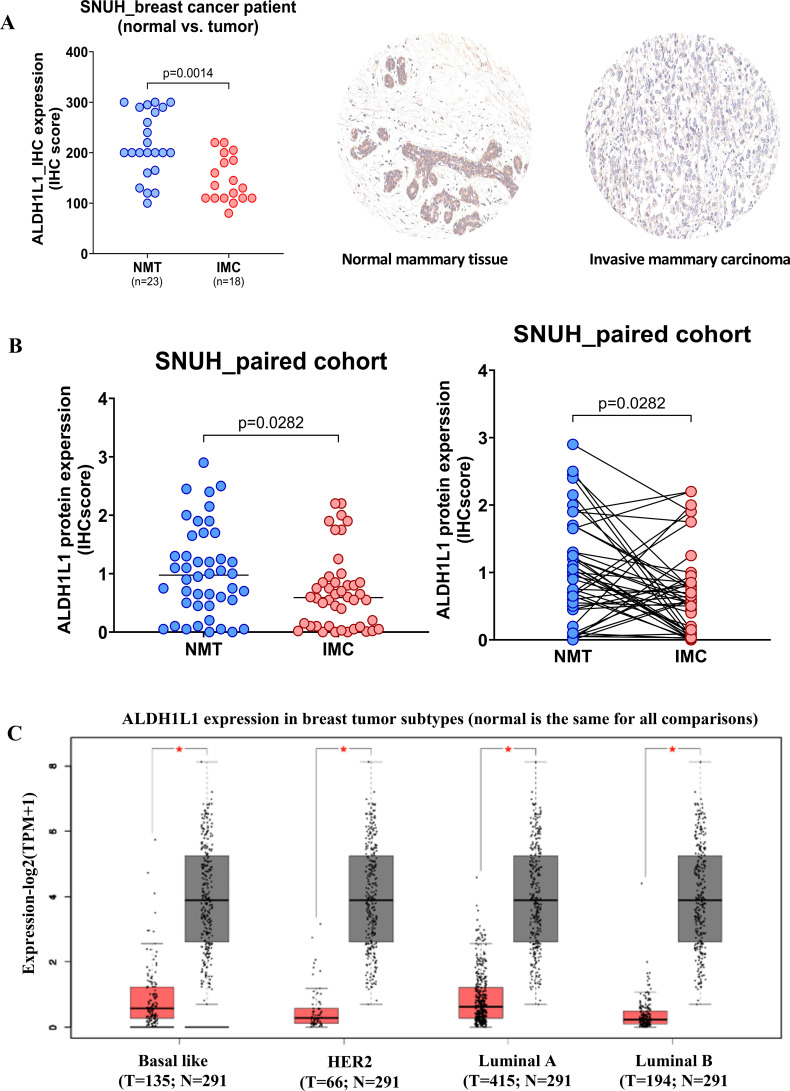
Expression level of ALDH1L1 in normal and cancer breast tissues. **(A)** ALDH1L1 protein expression evaluated by immunohistochemistry in non-tumor mammary glands (NMT, n = 23) and invasive mammary carcinoma (IMC, n = 18) patients (left panel). Representative immunohistochemical staining images in normal and breast cancer sections (right panels, x200). **(B)** ALDH1L1 protein expression in matched tissue samples (n = 44 pairs), in which normal mammary and breast cancer tissues were obtained from the same patient. Statistical analyses were performed using Student’s t-test. **(C)** Expression profiles of ALDH1L1 mRNA levels in all breast cancer subtypes and normal tissues (analyzed using the GEPIA2, http://gepia2.cancer-pku.cn/#index).

Since ALDH1L1 is considered a candidate tumor suppressor (35), we examined whether ALDH1L1 is differentially expressed between normal mammary tissue and mammary carcinoma. In our non-paired cohort of 41 patients, ALDH1L1 protein expression was significantly higher in non-tumorous mammary glands compared with mammary carcinoma cell clusters (Average H-score: 216.0 vs 146.1, NMT vs. IMC, p=0.0014, **Figure 1A**). Analysis of the second paired patient cohorts, which included matched normal and cancerous breast tissues (44 patients), showed consistently higher expression of ALDH1L1 protein in normal mammary glands compared to their cancerous counterparts (Average H-score: 102.6 vs 68.7, NMT vs. IMC, p=0.0282, **Figure 1B**). In agreement with our findings, we observed a statistically significant decrease in ALDH1L1 mRNA expression in breast cancer tissues compared to normal mammary tissues through analysis of publicly available databases, OncoDB (https://oncodb.org/) and GEPIA2 (http://gepia2.cancer-pku.cn/#index) (**Supplementary Figures 1A, B**). This analysis was conducted using the GEPIA2 web server, which enables comparison of gene expression between normal and tumor samples derived from the TCGA and GTEx datasets (39). We further evaluated the expression of mRNA for a set of folate-related proteins using GEPIA2. Remarkably, among all analyzed targets, ALDH1L1 was the most downregulated protein in breast cancer and the only downregulated cytosolic folate enzyme (**Supplementary Figure 2**). Of note, the downregulation of ALDH1L1 mRNA was observed in all breast cancer subtypes (**Figure 1C**).

### 3.3 Aggressive clinicopathologic parameters in breast cancer correlate with the loss of ALDH1L1 expression

**Figure 2:**
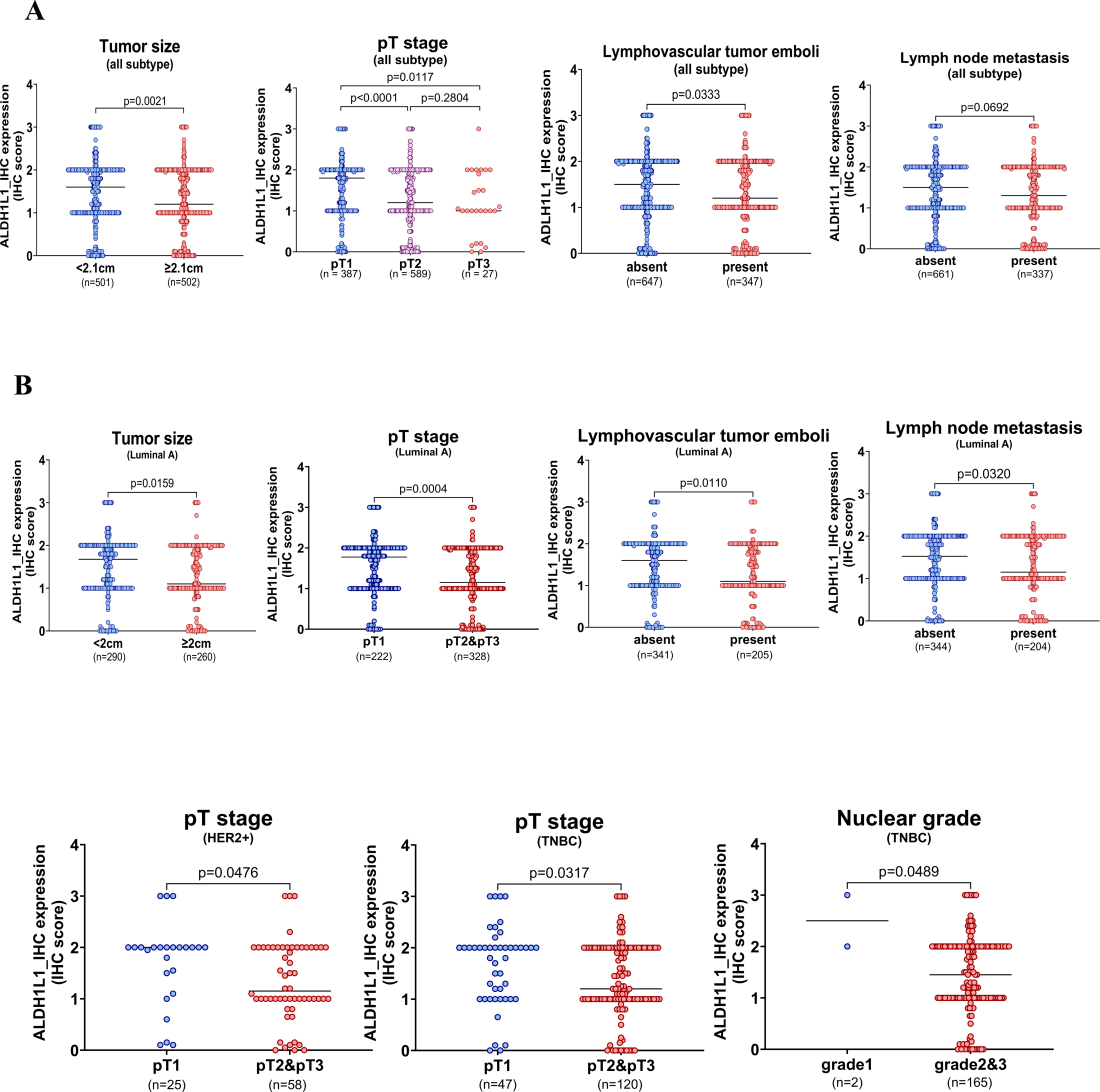
Correlation between clinicopathological parameters and ALDH1L1 expression levels in breast cancer patients. **(A)** Analysis of ALDH1L1 protein expression for different clinicopathological parameters across all breast cancer cases. **(B)** Same analysis for breast cancer subtypes (Luminal A, Luminal B, HER2+, and TNBC; from the SNUH cohort, including 1,001 patients). Associations between ALDH1L1 expression and clinicopathological variables were assessed using ANOVA and the Student’s *t*-test.

We further evaluated whether decreased expression of ALDH1L1 in breast cancer patients correlates with worsening clinicopathological parameters such as tumor grade and size, and lymphovascular tumor emboli (**Figure 2A**, **Supplementary Figure 3**). This analysis was performed for our third cohort, which included 1001 breast cancer patients. We found that the tumor size was larger in breast cancer patients with lower ALDH1L1 expression (p=0.0021). Further, as the pT stage advanced, the ALDH1L1 H-score decreased (p<0.0001). We also found that patients with lymphovascular tumor emboli had lower ALDH1L1 expression than patients without emboli (p=0.0333). Decreased ALDH1L1 expression showed a trend toward increased lymph node metastasis, although this association did not reach statistical significance (p=0.0692). This finding is consistent with the overall pattern linking low ALDH1L1 expression to aggressive clinicopathological features. Further analysis by intrinsic subtypes showed that the luminal A subtype with higher ALDH1L1 expression was associated with smaller tumor size (p=0.0159), lower pT stage (p=0.0004), reduced lymphovascular tumor emboli (p=0.0110), and a tendency for a lower rate of lymph node metastasis (p=0.0320, **Figure 2B**). In the HER2+ subtype, only pT stage showed a statistically significant association (p=0.0476), whereas in the TNBC subtype, pT stage (p=0.0317) and nuclear grade (p=0.0489) were significantly correlated (**Figure 2B**, **Supplementary Figure 3**).

### 3.4 Prognostic implication of ALDH1L1 in breast cancer

**Figure 3:**
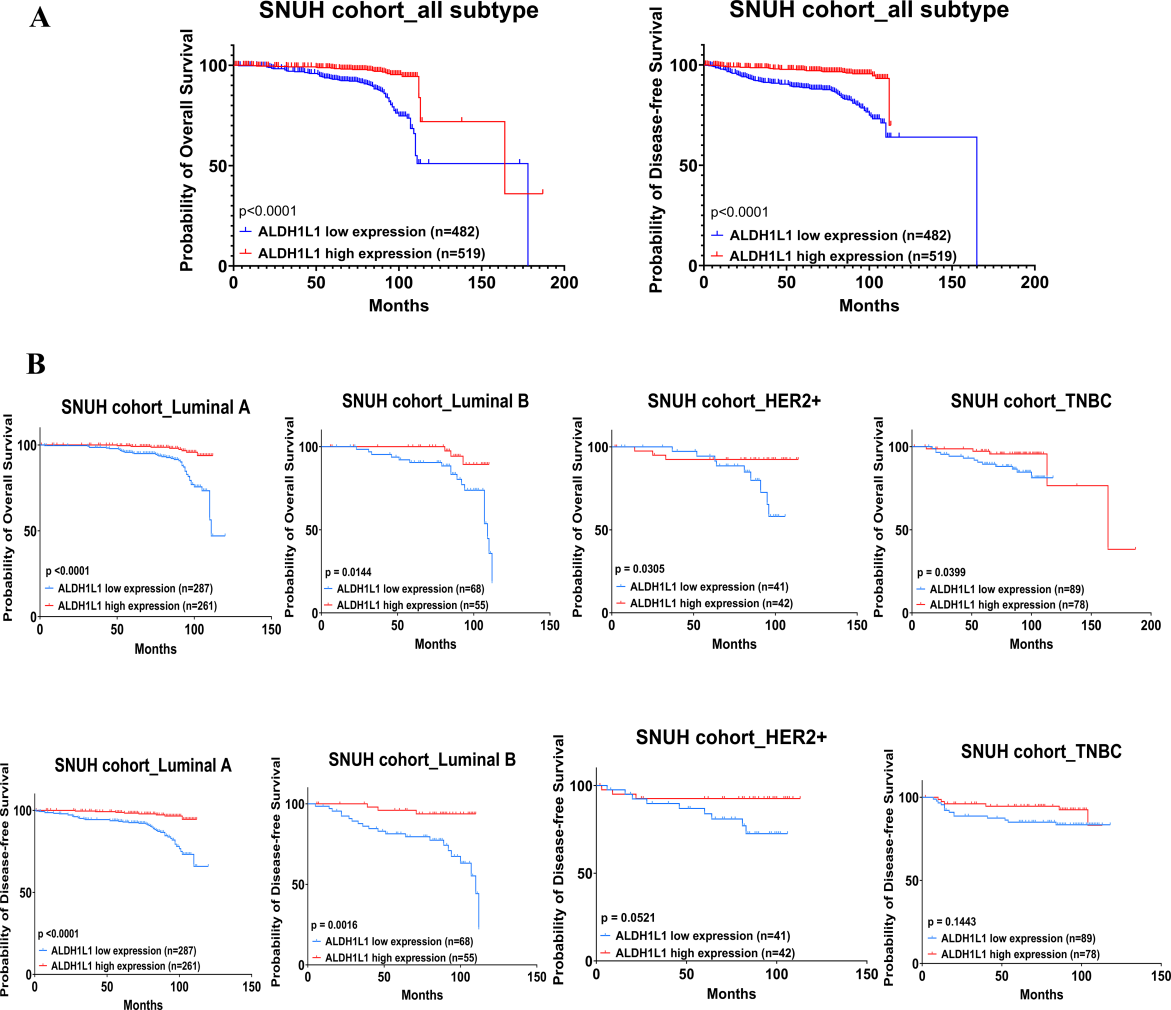
Comparative analysis between ALDH1L1 expression and prognosis in breast cancer patients. Kaplan-Meier survival analysis of the entire SNUH breast cancer cohort **(A)** and across breast cancer subtypes **(B)**. Analysis was stratified by high and low ALDH1L1 expression groups for the overall and disease-free survival using an H-score of 210 as the cut-off.

We assessed the clinical significance of ALDH1L1 expression by analyzing our largest breast cancer patient cohort (1001 patients). ALDH1L1 was expressed (H-score cut-off: 1.3, 519/1,001) and strongly associated with prolonged overall (OS) as well as disease-free (DFS) survival (OS p<0.0001; DFS p<0.0001, **Figure 3A**). Interestingly, increased ALDH1L1 expression was associated with favorable OS and DFS compared with low ALDH1L1 expression in HR-positive patients. Thus, survival for patients with high vs. low ALDH1L1 based on the subtype, was as follows. OS luminal A: p<0.0001; DFS luminal A: p<0.0001; OS luminal B: p=0.0144; DFS luminal B: p=0.0016 (**Figure 3B**). However, there was only marginal (not statistically significant) prognostic relevance of ALDH1L1 in HR-/HER2+ and HR-/HER2- patients (HER2, OS p=0.0305 and DFS p=0.0521; TNBC, OS p=0.0399 and DFS p=0.1443; **Figure 3B**).

**Figure 4:**
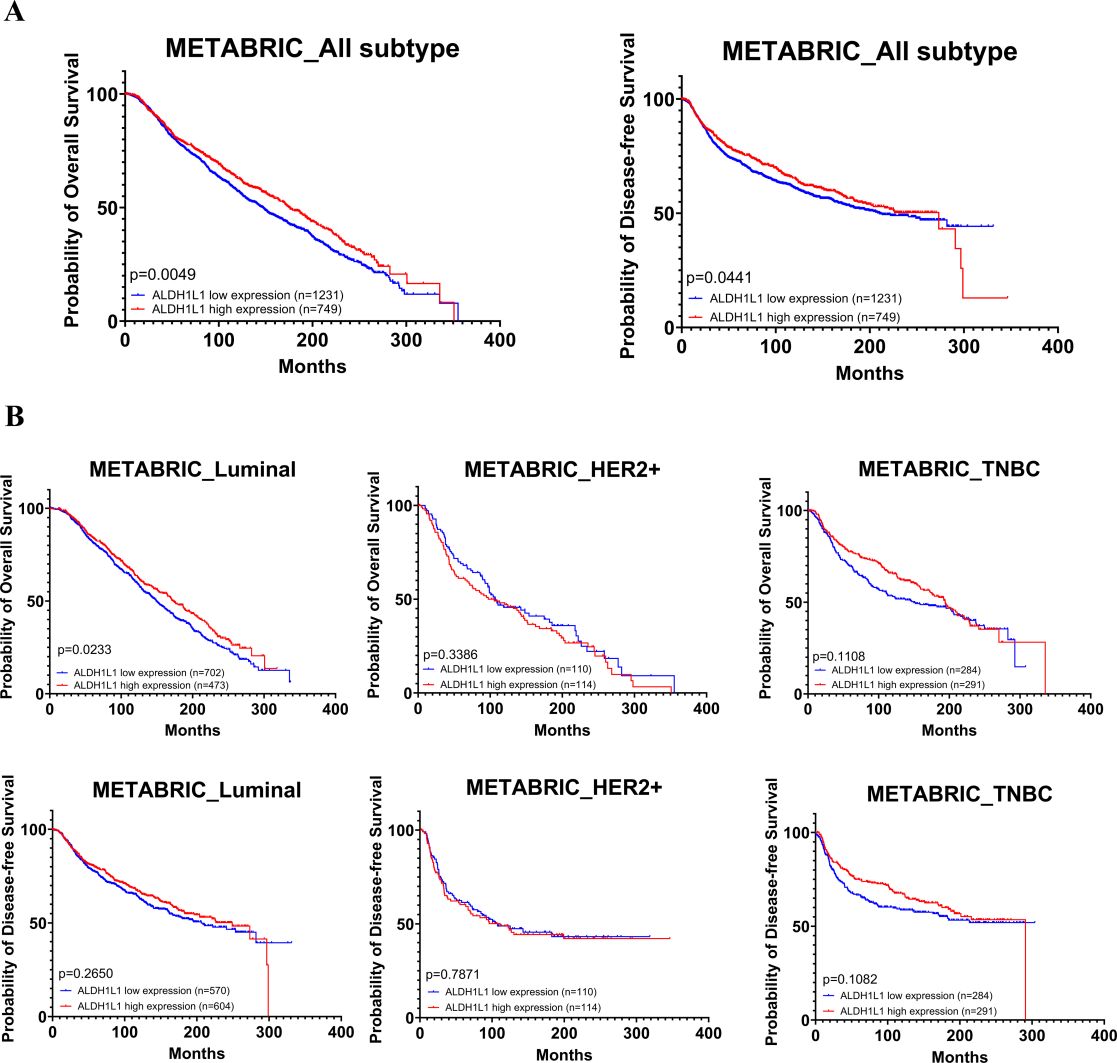
Validation of ALDH1L1 expression as the prognostic marker in breast cancer patients (data from the METABRIC database were analyzed). **(A)** all breast cancer patients in the cohort and **(B)** across breast cancer subtypes: Luminal A, Luminal B, HER2+, and TNBC patients (METABRIC, n=1,980). Overall survival (OS) and disease-free survival (DFS) analyses were conducted using the Kaplan–Meier curves, and different groups were compared using a log-rank test.

To further validate the prognostic significance of ALDH1L1 expression, we used the METABRIC database to analyze mRNA expression data for ALDH1L1 across a cohort of 1,980 breast cancer samples. This analysis indicated that higher ALDH1L1 expression was associated with prolonged OS (p=0.0049, **Figure 4A**). Although DFS appeared to be poorer with high ALDH1L1 expression (p=0.0441), the final survival point showed that patients with high ALDH1L1 expression had a longer median survival (346.38 months) compared to those with low expression (331.18 months, **Figure 4A**). Consistent with the findings from our breast cancer cohort, ALDH1L1 was significantly associated with OS (p=0.023) but had a tendency for better DFS (p=0.265) in the luminal breast cancer subtype (**Figure 4A**). No predictive role of ALDH1L1 was found in other subtypes, including HER2-positive or TNBC (**Figure 4B**).

To determine whether ALDH1L1 expression represents an independent prognostic factor in breast cancer, we performed Cox proportional hazards regression analysis, adjusting for age, T stage, N stage, histologic grade, lymphatic invasion, and Ki-67 index. In multivariate analysis, high ALDH1L1 expression remained significantly associated with improved overall survival (HR = 0.22, 95% CI 0.13–0.35, p<0.001; **Table 3**). Similar results were observed for disease-free survival, where ALDH1L1 expression was independently associated with a reduced risk of recurrence (HR = 0.22, 95% CI 0.14–0.35, p<0.001; **Table 4**).

**Table 3:** Multivariate Cox regression analysis for overall survival.

Variables	HR	95% CI	p-value
ALDH1L1 expression			
High vs low	0.22	0.13-0.35	<0.001
Age (years)			
≥ 50 vs < 50	1.42	0.96-2.10	0.082
T stage			
Stage II vs Stage I	1.37	0.82-2.28	0.23
Stage III vs Stage I	4.0	1.66-9.63	0.002
N stage			
Stage I vs Stage 0	2.08	1.33-3.27	0.001
Stage II vs Stage 0	1.06	0.5-2.23	0.883
Stage III vs Stage 0	4.44	2.14-9.22	<0.001
Histologic grade			
Grade 2 vs Grade 1	2.32	0.31-17.3	0.41
Grade 3 vs Grade 1	4.85	0.66-35.5	0.12
Lymphatic invasion			
Present vs Absent	1.34	0.88-2.04	0.177
Ki67			
% increase	1.02	1.0-1.03	0.019

**Table 4:** Multivariate Cox regression analysis for disease-free survival.

Variables	HR	95% CI	p-value
ALDH1L1 expression			
High vs low	0.22	0.14-0.35	<0.001
Age (years)			
≥ 50 vs < 50	1.44	0.99-2.11	0.056
T stage			
Stage II vs Stage I	1.37	0.85-2.21	0.199
Stage III vs Stage I	3.4	1.46-7.94	0.005
N stage			
Stage I vs Stage 0	2.0	1.3-3.08	0.002
Stage II vs Stage 0	1.15	0.57-2.34	0.69
Stage III vs Stage 0	4.4	2.21-8.75	<0.001
Histologic grade			
Grade 2 vs Grade 1	1.42	0.34-5.98	0.636
Grade 3 vs Grade 1	2.69	0.65-11.1	0.172
Lymphatic invasion			
Present vs Absent	1.6	1.06-2.41	0.025
Ki67			
% increase	1.01	1.0-1.03	0.033

### 3.5 Correlations between expression of ALDH1L1 and markers for proliferation and tumorigenesis

We analyzed the correlation between ALDH1L1 expression and Ki-67, a well-established marker of tumor cell proliferation, in our large SNUH TMA cohort comprising 1,001 invasive ductal carcinoma cases. We observed a negative correlation between ALDH1L1 and Ki-67 expression across the entire cohort (**Supplementary Figure 4A**). When stratified by breast cancer subtype, these associations did not reach statistical significance (**Supplementary Figure 4**). Although subtype-specific correlations did not consistently reach statistical significance, the inverse trends were more evident in luminal tumors, particularly luminal B, compared with HER2-positive or TNBC subtypes. This pattern may reflect the hormone receptor–positive and relatively differentiated molecular characteristics of luminal cancers, which are generally associated with more regulated proliferative activity. Given that high expression of basal markers such as Cytokeratin 5/6 (CK5/6) has also been associated with poor clinical outcomes in breast cancer (40, 41), we additionally investigated the relationship between ALDH1L1 expression and CK5/6 to further elucidate the link between ALDH1L1 and aggressive tumor phenotypes. We observed a weak but statistically significant negative correlation between ALDH1L1 and CK5/6 expression in the luminal A and HER2+ subtypes, whereas no significant associations were detected in other subtypes (**Supplementary Figure 5**). This additional finding further reinforces the tumor-suppressive role of ALDH1L1 in breast cancer, supporting its inverse association with markers of aggressive tumor phenotypes.

**Figure 5:**
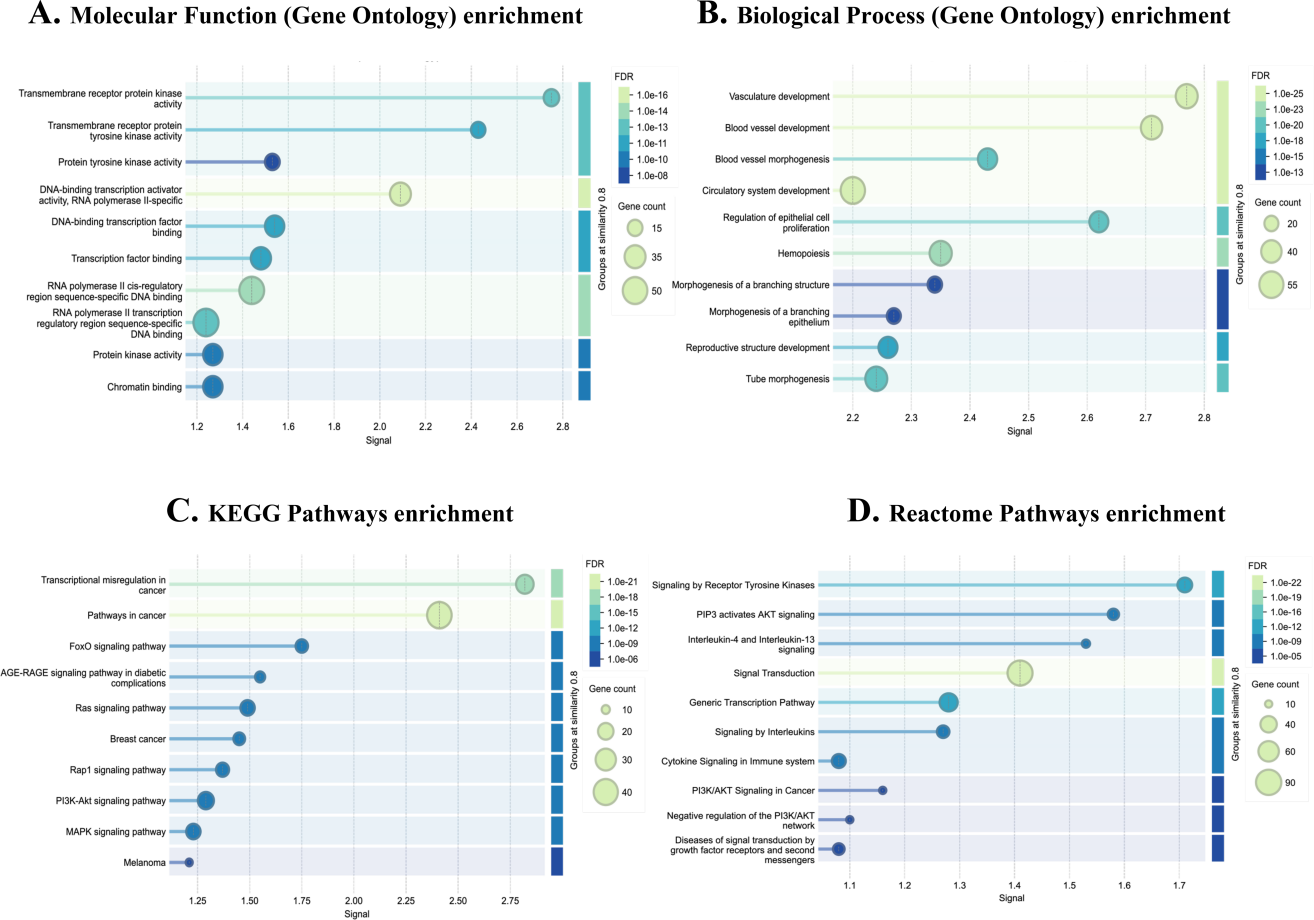
Gene ontology (GO) and pathway enrichment annotation for genes correlated with ALDH1L1 expression in breast cancer. Combined set of genes from **Supplementary Table 1** and S2 was analyzed using corresponding modules in the STRING database (https://string-db.org/). Bubble plots display significantly enriched terms across four categories: **(A)** biological processes enrichment; **(B)** molecular function enrichment; **(C)** KEGG pathways enrichment; **(D)** reactome pathways enrichment. Bubble size represents the number of genes associated with each term, and color intensity reflects statistical significance (false discovery rate, FDR).

Based on the METABRIC database, we analyzed representative markers of tumor cell invasiveness, metastasis, proliferation, and recurrence. We found that most of these markers showed a negative correlation with ALDH1L1 expression, which is consistent with the results of our study. Moreover, not only in the luminal A subtype but also in other breast cancer subtypes, most markers of proliferation, metastasis, and recurrence exhibited a negative correlation with ALDH1L1 expression (**Supplementary Table 1**). Additional correlation analysis was performed for breast cancers from the TCGA database using the GEPIA2 online platform. Genes for analysis were selected based on reported markers of breast cancers (42, 43), and the OncoKB™ Cancer Gene List platform (https://www.oncokb.org/) (44). Overall, we have analyzed the correlation between ALDH1L1 expression and the expression of more than 1,216 genes, which were linked to breast cancer. Of the 1,216 analyzed oncogenes, 193 were found to be significantly associated with ALDH1L1 expression in BRCA tumors (**Supplementary Table 2**). We have observed strong correlations, both negative and positive, between ALDH1L1 expression and several well-established markers of breast cancer, including estrogen receptors, BRCA1, Ki-67, TGFbR2, PAI1, and FOXO1 (**Supplementary Table 2**). To assess cellular processes linked to ALDH1L1 function in breast cancer, we analyzed the combined set of genes from **Supplementary Tables 1**, **S2** using the STRING database (45). The application of two gene ontology modules within this platform, Molecular Function Enrichment and Biological Process Enrichment, indicated that ALDH1L1 expression correlates with expression of genes involved in cancer signaling and transcriptional regulation (**Figures 5A, B**). The enrichment analysis (**Figures 5C, D**) highlighted enhanced representation of genes involved in extracellular matrix organization, cell adhesion and cell migration, which further indicates the association between ALDH1L1 expression and pathways governing tumor cell motility and tissue remodeling. Overall, our analysis indicates that ALDH1L1 expression is linked to the gene network associated with mammary tumorigenesis and tumor progression. Though our analysis has limitations for the precise interpretations of the observed correlation because all cancer subtypes were analyzed as a single group, it provides further support for the role of ALDH1L1 in breast cancer. While our current study focused on the luminal A subtype, these findings suggest the importance of extending future investigations to other breast cancer subtypes to validate and expand the clinical significance of ALDH1L1.

### 3.6 Antiproliferation and antimigratory effects of ALDH1L1 in hormone receptor-positive breast cancer cells

**Figure 6:**
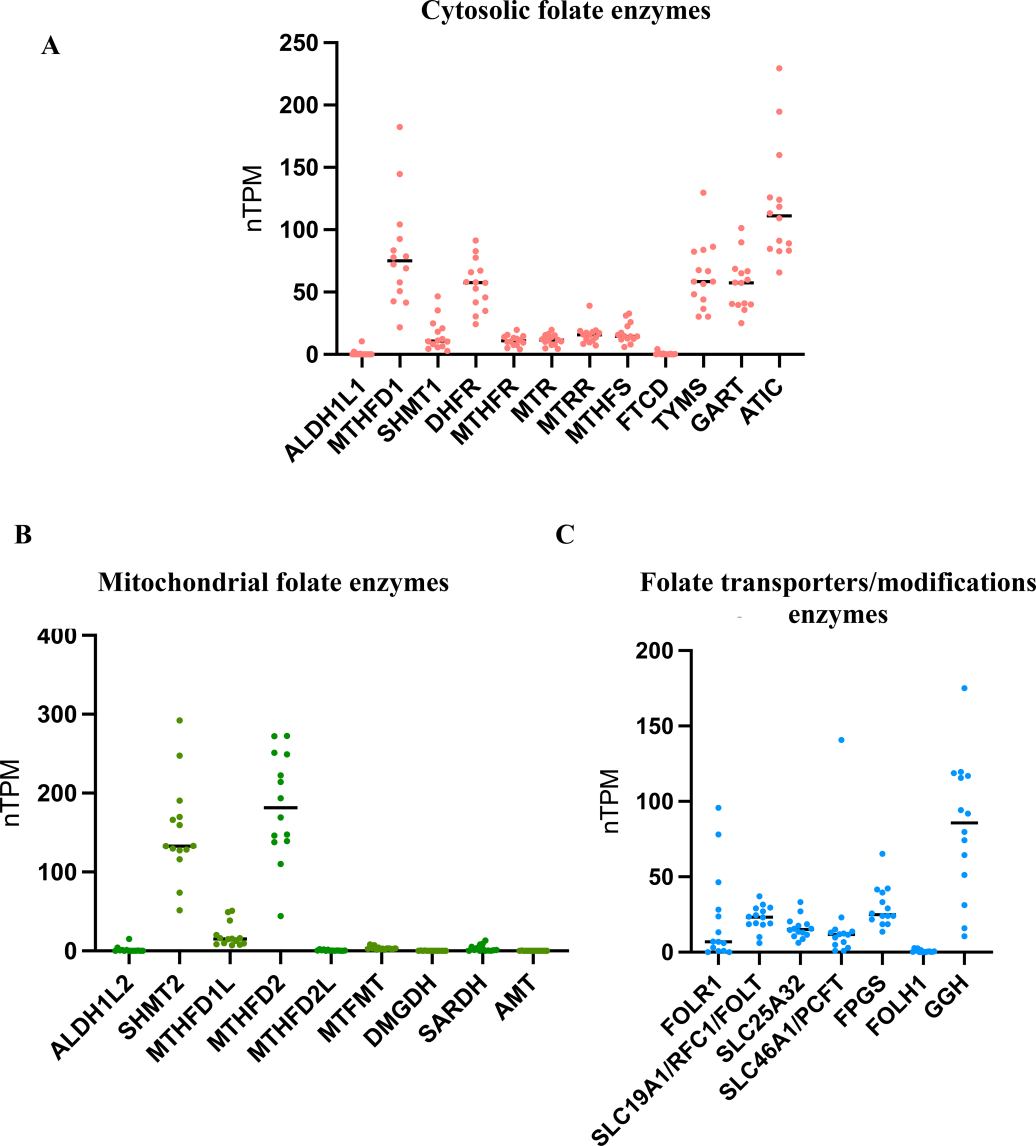
Expression of folate enzymes (mRNA) in a panel of 13 luminal breast cancer cell lines based on the human protein atlas (https://www.proteinatlas.org/). Levels of mRNA for cytosolic folate enzymes **(A)**, mitochondrial folate enzymes **(B)** and folate transporters/enzymes involved in folate polyglutamylation **(C)**; each dot represents the mRNA levels for a single cell line; cell lines are listed in **Supplementary Table 3**. Abbreviations for protein names are based on the gene nomenclature as depicted in the Human Protein Atlas.

**Figure 7:**
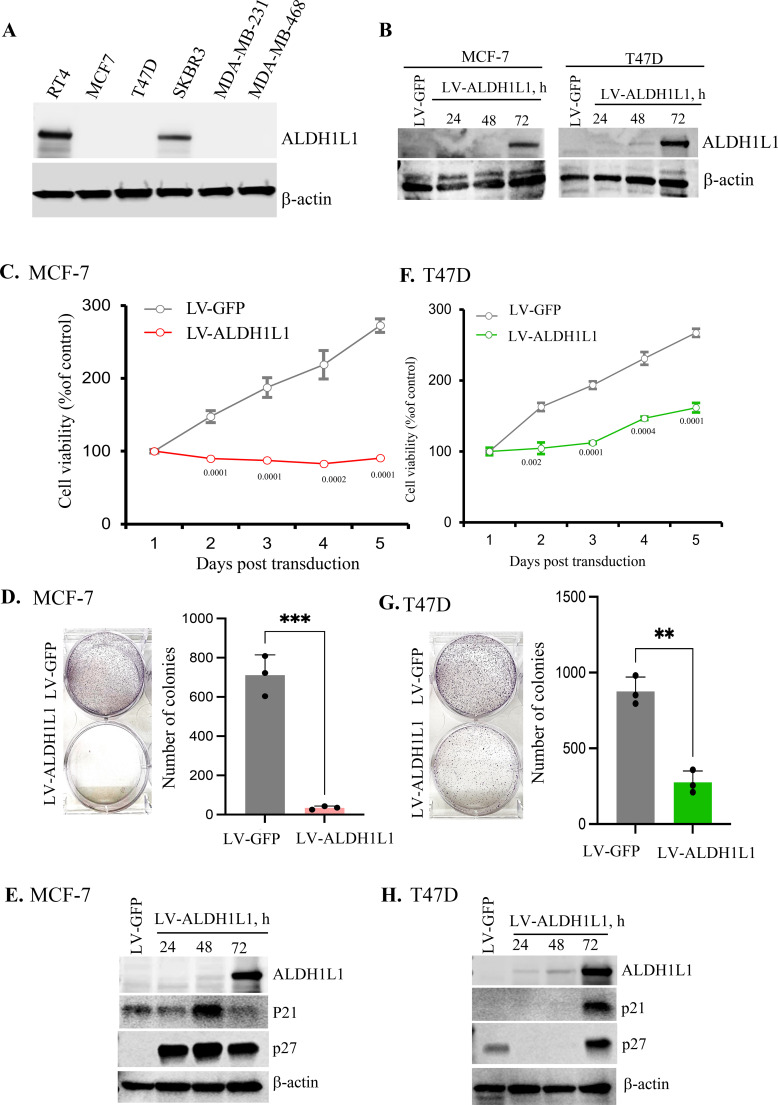
Effect of ALDH1L1 knock-in on cellular proliferation of hormone receptor-positive breast cancer cell lines. **(A)** ALDH1L1 immunoblot assay in several breast cancer cell lines representing luminal A, luminal B, HER-2, and TNBC subtypes. Full-size blot is shown in **Supplementary Figure 7A**. **(B)** Levels of ALDH1L1 in MCF7 (*left panel*) and T47D (*right panel*) cells (immunoblot assay) at different time points post-transduction. Full-size blot is shown in **Supplementary Figure 7B**. **(C)** Proliferation of MCF7 cells expressing ALDH1L1 or GFP (control). Proteins were expressed by lentiviral delivery. Data presented as means ± SD (n=3). **(D)** Colony formation of ALDH1L1-expressing MCF7 cells compared to GFP-expressing control cells after 14 days of incubation. Quantification of colony numbers means ± SEM (n=3). ***p<0.0001; **p<0.001. **(E)** Immunoblot analysis of p21 and p27 expression in ALDH1L1-transduced MCF7 cells. **(F-H)** Proliferation, colony formation, and immunoblot analysis of p21 and p27 in T47D cells. Full-size blot is shown in **Supplementary Figures 8A–D**.

In agreement with the findings in our human breast cancer cohorts and publicly available databases, we also observed that ALDH1L1 was among the most underexpressed folate-related proteins and the most underexpressed cytosolic folate enzyme in luminal breast cancer cells (**Figures 6A–C**). This analysis was performed using data from the Human Protein Atlas (the list of analyzed cell lines is shown in **Supplementary Table 3**). We further analyzed ALDH1L1 protein expression in a set of breast cancer cell lines representing different breast cancer subtypes (MCF7, luminal A; T47D, luminal B; SKBR3, HER2 subtype; MDA-MB-231 and MDA-MB-468, triple-negative subtype). SKBR3 cells exhibited a moderate level of ALDH1L1 expression among all tested cell lines, whereas the other four cell lines showed no detectable expression of the protein (**Figure 7A**; RT4, a bladder cancer cell line with high ALDH1L1 expression (46), was used as a positive control). These findings were further supported by analysis of ALDH1L1 mRNA expression across the same cell lines using the Human Protein Atlas database (**Supplementary Figure 6**).

**Figure 8:**
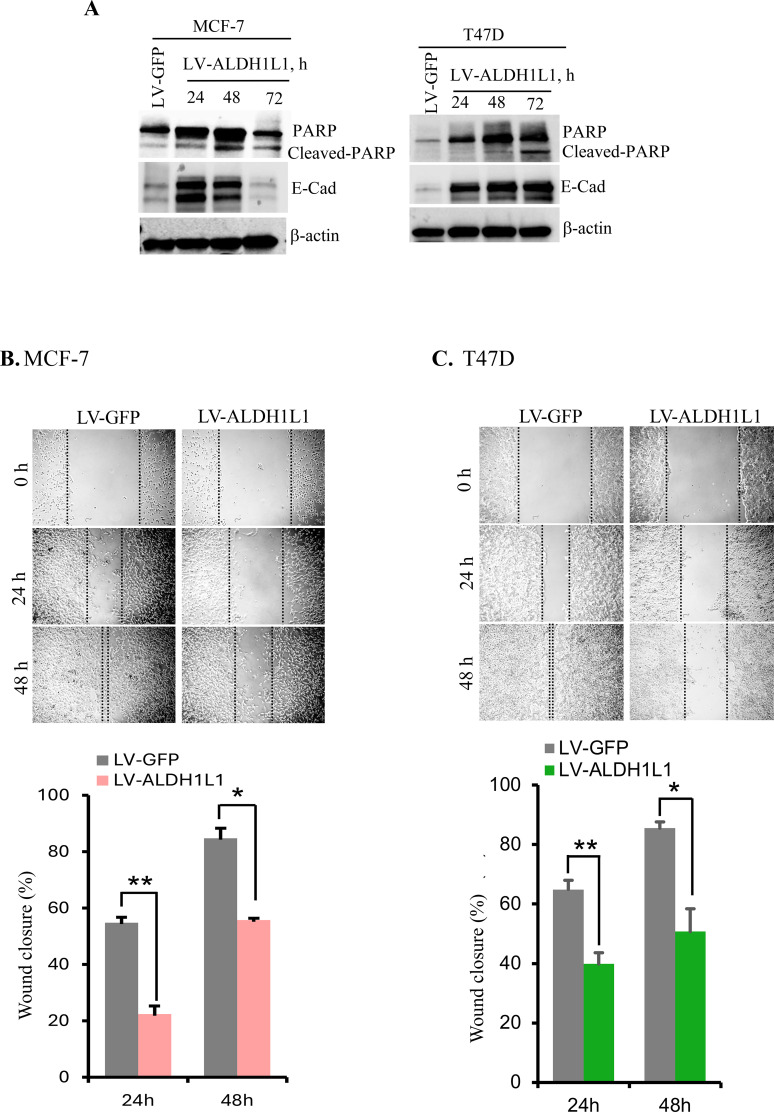
Effect of ALDH1L1 knock-in on cell motility of hormone receptor-positive breast cancer cell lines. **(A)** Immunoblot analysis of PARP, cleaved PARP, and E-Cadherin expression in ALDH1L1-transduced MCF7 (*Left panel*) and T47D (*Right panel*) cells. Full-size blot is shown in **Supplementary Figures 9A–C**. **(B)** Upper panels, wound healing assay images showing motility of ALDH1L1-transduced MCF7 cells over a 24 and 48 h period. Lower panel, bar graph representing the mean ± SEM of wound closure at 24 and 48 h measured in triplicate. **(C)** Upper panels, wound healing assay images showing motility of ALDH1L1-transduced T47D cells over a 24 and 48 h period. Lower panels, bar graph representing the mean ± SEM of wound closure at 24 and 48 h measured in triplicate. Data show mean ± SEM; **p < 0.001; *p < 0.05.

Next, we investigated the antiproliferative function of ALDH1L1 in breast cancer cells. Based on our clinical data, which indicated that the luminal subtype has a more promising clinical relevance for ALDH1L1 expression, we selected luminal breast cancer cell lines MCF7 and T47D for these studies. Since these cells do not express ALDH1L1, we knocked in the protein using the lentiviral vector delivery. In both cell lines, a robust expression of ALDH1L1 was achieved 72 h after lentiviral transduction with an MOI of 50 (**Figure 7B**). We observed that ALDH1L1 expression strongly inhibited both proliferation and the clonogenic capacity of MCF7 cells (**Figures 7C, D**). In agreement with these effects, levels of two proliferation regulators, p21 and p27, were significantly elevated in response to ALDH1L1 expression (**Figure 7E**). These two proteins are known to restrict proliferation by inducing cell cycle arrest at the G1/S transition step (47). Similar effects of ALDH1L1 expression on proliferation, clonogenic capacity, and the p21/p27 induction were observed in T47D cells (**Figures 7F-H**). Further, in both cell lines, ALDH1L1 expression led to enhanced PARP cleavage 48–72 h post transduction (**Figure 8A**), the finding indicative of the activation of apoptotic pathways. A pronounced elevation of E-cadherin was also observed in MCF7 and T47D cells at 24–48 h and 24–72 h ALDH1L1 post-transduction, respectively, compared to control GFP-transduced cells (**Figure 8A**), which suggests inhibition of the epithelial–mesenchymal transition (EMT). Given the importance of EMT for enabling migration and invasion of cancer cells, we next examined the effect of ALDH1L1 on cell motility. Wound healing assays have shown that ALDH1L1-expressing cells have markedly suppressed motility, whereas ALDH1L1-deficient cells displayed high migratory activity (**Figures 8B, C**). Of note, the effect of ALDH1L1 expression on motility was similar in both MCF7 and T47D cell lines.

## 4 Discussion

In this study, we investigated the clinical role of ALDH1L1 as a novel biomarker in patients with breast cancer. Our findings indicate that the expression of ALDH1L1 is significantly suppressed in breast cancer tissues compared to normal tissues, which is consistent with previous reports for several cancer types, including breast cancer (14, 16, 18, 48, 49). Overall, there is a consensus in the literature that reduced ALDH1L1 expression is associated with increased tumor growth and progression (reviewed in (19, 28)). This was likely due to the ability of this enzyme to regulate cellular proliferation (50). In fact, ALDH1L1 is tightly controlled by ubiquitination and proteasomal degradation during the cell cycle progression (51). Thus, in NIH3T3 cells, the enzyme is not detected in the S-phase of the cell cycle when cells are actively prepared for division but is highly expressed in quiescent cells (51). In several cancers, the downregulation of ALDH1L1 is linked to promoter methylation (14–18), a mechanism that produces sustained downregulation of gene expression. In mammary gland tissue, the promoter methylation of *ALDH1L1* has also been suggested as a key mechanism of this gene regulation (52, 53). Higher CpG methylation in *ALDH1L1* significantly correlated with lower mRNA levels of the protein in normal breast tissue, as well as in breast cancer (18, 52). Interestingly, differential methylation of *ALDH1L1* in normal breast tissues was observed in a study that included 81 participants with no history of reduction mammoplasty due to breast cancer (52). This raises the question of whether lower ALDH1L1 expression in normal breast tissue could be a predisposition for malignant transformation.

Our analysis revealed that breast cancer patients with lower ALDH1L1 expression exhibited reduced survival rates compared to those with higher ALDH1L1 expression. At the protein level, ALDH1L1 was undetectable by western blot assays in established cancer cell lines but was expressed in non-cancerous immortalized cell lines (13, 51). These findings are in agreement with our analysis of the CCLE dataset, which showed approximately two-fold higher ALDH1L1 mRNA expression in immortalized non-cancerous mammary cell lines than in cell lines derived from malignant breast tumors. Importantly, our correlation analysis aligns with our overall findings that ALDH1L1 exerts tumor-suppressive effects, as evidenced by its association with less aggressive clinicopathologic features and reduced proliferation in functional assays. While the prognostic role of ALDH1L1 in breast cancer has been previously evaluated (29), earlier conclusions may be limited by analytical resolution. Results of the present study refine and, in part, revise earlier observations due to improved dataset composition and more precise molecular subtype-specific analysis.

Our analysis by intrinsic subtypes showed a strong association between clinical outcomes and ALDH1L1 mRNA and protein expression levels, particularly in HR-positive breast cancer, suggesting its potential role as a biomarker for this subtype. Several studies have shown that purine biosynthesis regulates ERα activity by sustaining nucleotide pools and activating downstream signaling pathways that promote tumor growth. This metabolic reprogramming has been implicated in driving therapeutic resistance in HR-positive breast cancers, underscoring the link between altered metabolism and endocrine therapy failure (54, 55). Of note, our analysis indicated a negative correlation between expression of ERa and ALDH1L1 in breast cancer. Since ALDH1L1 reaction competes with the *de novo* purine biosynthesis for the same substrate, 10-formyltetrahydrofolate, the downregulation of the enzyme could be a mechanism by which cancer cells increase the flow of one-carbon groups towards nucleotide generation. In addition, our *in-silico* analysis revealed that ALDH1L1 expression is consistently associated with key components of the folate-mediated one-carbon metabolic network. Notably, ALDH1L1 showed negative correlations with genes involved in folate-dependent nucleotide biosynthesis, including MTHFD1, MTHFD2, DHFR, and TYMS, while positive correlations were observed with serine biosynthesis–related genes such as PHGDH and PSAT1. These findings are mechanistically consistent with the enzymatic role of ALDH1L1 in depleting one-carbon units from the folate pool, thereby potentially limiting nucleotide synthesis and cellular proliferation. Accordingly, upregulation of ALDH1L1 in malignant tumors produces a metabolic effect similar to that of antifolate drugs, leading to the inhibition of cellular proliferation (19). In agreement with such a mechanism, our previous studies have demonstrated the impact of ALDH1L1 on intracellular purine nucleotide pools (56, 57). These findings raise the possibility that ALDH1L1 expression could influence responses to purine-targeting agents (e.g., 6-mercaptopurine, 6-thioguanine) and to the thymidylate synthase inhibitor 5-fluorouracil (58–61). Importantly, given that the prognostic impact of ALDH1L1 was most evident in luminal breast cancer, its expression may have particular clinical relevance in this subtype. Luminal tumors are primarily treated with endocrine therapy, often in combination with chemotherapy, depending on recurrence risk. Therefore, stratifying patients according to ALDH1L1 expression may help refine risk assessment and potentially inform therapeutic decision-making, especially in cases where chemotherapy is being considered.

Collectively, our findings suggest that ALDH1L1 may serve as a subtype-specific biomarker in luminal breast cancer, warranting further investigation in treatment-stratified cohorts. In contrast, the associations between ALDH1L1 expression and survival outcomes in HER2+ and TNBC cohorts were marginal or not statistically significant. Although ALDH1L1 expression was consistently lower in tumors compared with normal breast tissue across all subtypes, a survival advantage of high ALDH1L1 expression was observed only in luminal cases. A limitation of the present study is that, although reduced ALDH1L1 expression was associated with poorer survival in both luminal A and luminal B tumors, corresponding clinicopathological differences were not observed in the luminal B subgroup. This discrepancy may reflect the biological heterogeneity of breast cancer and suggests that conventional clinicopathological variables do not fully capture the prognostic information associated with ALDH1L1 expression. These findings indicate that the prognostic value of ALDH1L1 is subtype-specific, and additional studies are needed to define its precise role in different breast cancer contexts. The subtype-specific patterns of ALDH1L1 expression and their associations with clinicopathologic features are summarized in **Table 5**.

**Table 5:** Subtype-specific associations of ALDH1L1 expression with clinicopathologic features and survival outcomes in breast cancer.

Variables	Luminal A	Luminal B	HER2+	TNBC
ALDH1L1 expression level	High	Moderate/Low	Low	Low
Lymph node metastasis	Inverse	Positive association	Positive association	Inverse
Ki-67 correlation	Inverse	Inverse	Weak	Weak
Survival impact	Most significant	Significant	Not significant	Not significant

**Figure 9:**
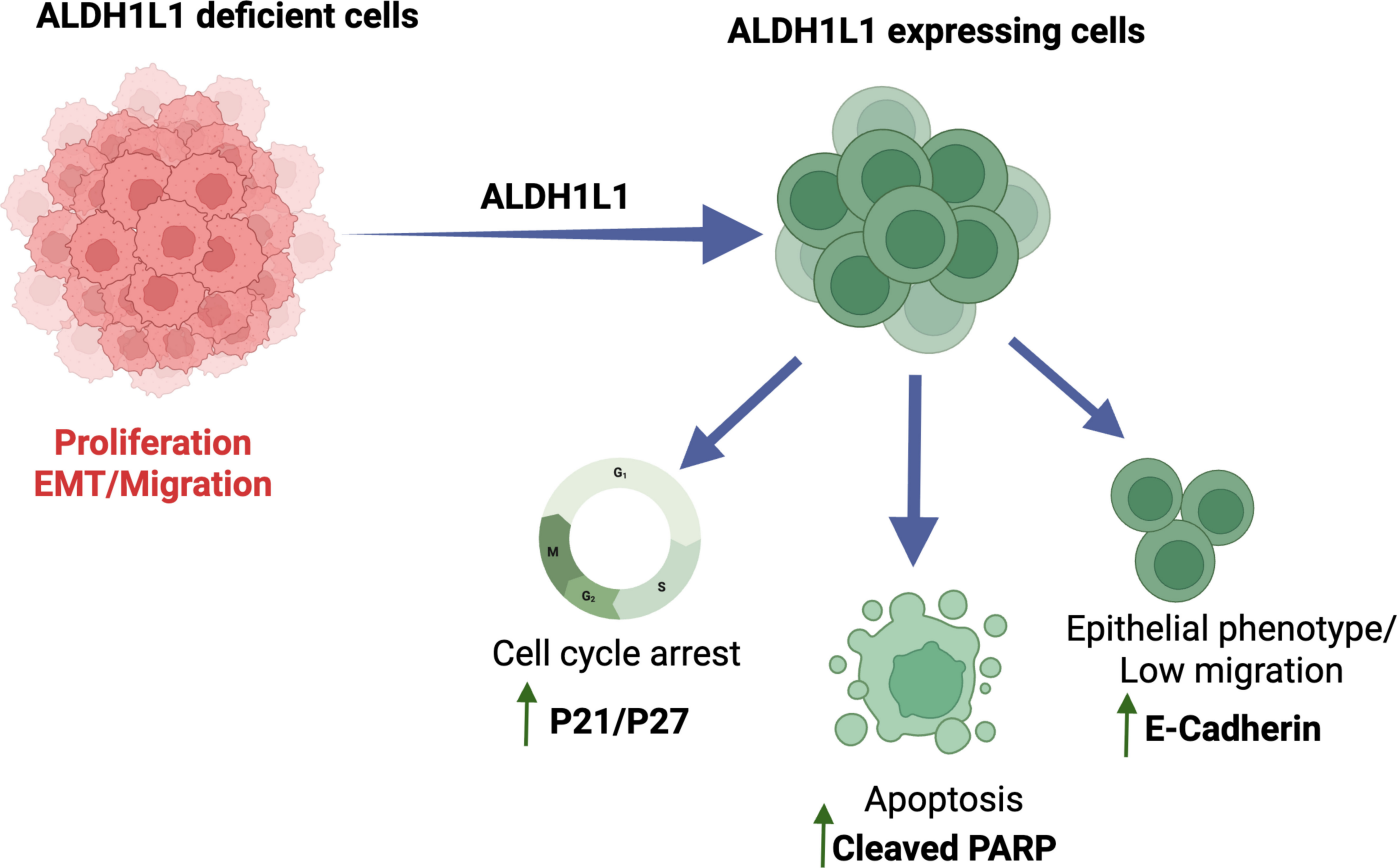
Effects of ALDH1L1 function in luminal breast cancer cells (schematic was created using BioRender).

The present study supports the role of the metabolic regulator and a candidate tumor suppressor ALDH1L1 in breast cancer progression and resistance to chemotherapies targeting certain metabolic pathways. Functionally, ALDH1L1 expression could curtail the growth potential of luminal breast cancer cells by engaging multiple anti-proliferative pathways, including the reactivation of cell cycle checkpoints and the induction of apoptotic signaling (**Figure 9**). This model is consistent with prior reports of ALDH1L1 acting as a metabolic brake on proliferation signaling in malignancies (13, 35, 37, 46, 56, 57, 62–66), the mechanism likely underlying the supposed role of the enzyme as a tumor suppressor. A particularly notable observation is the effect of ALDH1L1 on E-cadherin, which induction is commonly linked to the epithelial phenotype and suppressed EMT. Since EMT is required for cancer cell migration and is widely recognized as a driver of metastasis (67, 68), ALDH1L1 may contribute to limiting both local tumor expansion and metastatic dissemination. In support of such a possibility, we have previously reported the effect of ALDH1L1 on cell motility (64). Thus, ALDH1L1-mediated metabolic control of invasion and migration in luminal breast cancers represents an underexplored mechanism, which is at the intersection of metabolic reprogramming, cell cycle control, apoptosis, and epithelial plasticity (**Figure 9**).

## 5 Conclusion

Overall, this study identifies ALDH1L1 as a clinically relevant biomarker of breast cancer progression and patient survival. Our findings suggest that ALDH1L1 may represent a promising therapeutic target for precision medicine approaches, particularly in luminal breast cancers where its prognostic and functional significance appears most pronounced. In contrast, the prognostic and therapeutic value of ALDH1L1 in HER2+ and TNBC subtypes remains unclear. Additional limitation of the study is that significant differences in overall survival in luminal B tumors were observed despite the lack of statistically significant associations with clinicopathological parameters. This apparent discrepancy may reflect the fact that conventional clinicopathological variables do not fully capture the biological complexity of tumors. It is possible that ALDH1L1 expression reflects molecular or metabolic features that influence patient outcomes independently of these parameters. Therefore, this finding does not contradict our results but rather suggests that ALDH1L1 may provide additional prognostic information beyond conventional clinicopathological characteristics. To validate our observations and better define the mechanistic role of ALDH1L1 in breast cancer biology, future studies with larger and independent patient cohorts will be essential. Such efforts may ultimately clarify the translational potential of ALDH1L1 both as a biomarker and therapeutic target in subtype-specific breast cancer management.

## Data Availability

The original contributions presented in the study are included in the article/**Supplementary Material**. Further inquiries can be directed to the corresponding author.
