# Supplementary material for: Clinical implications of 10-formyltetrahydrofolate dehydrogenase expression in hormone receptor-positive breast cancer
Source: Front Oncol. 2026 May 25;16:1838093. doi: 10.3389/fonc.2026.1838093 (PMC13243080; doi:10.3389/fonc.2026.1838093)
Supplement: Supplementary file 1 [file DataSheet1.pdf]

Supplementary Figure 1

A

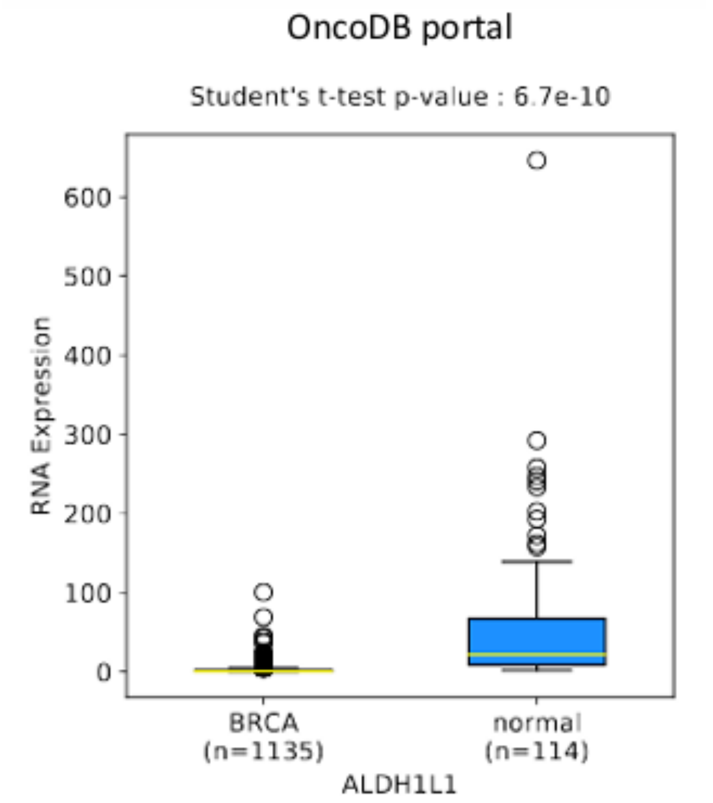

B

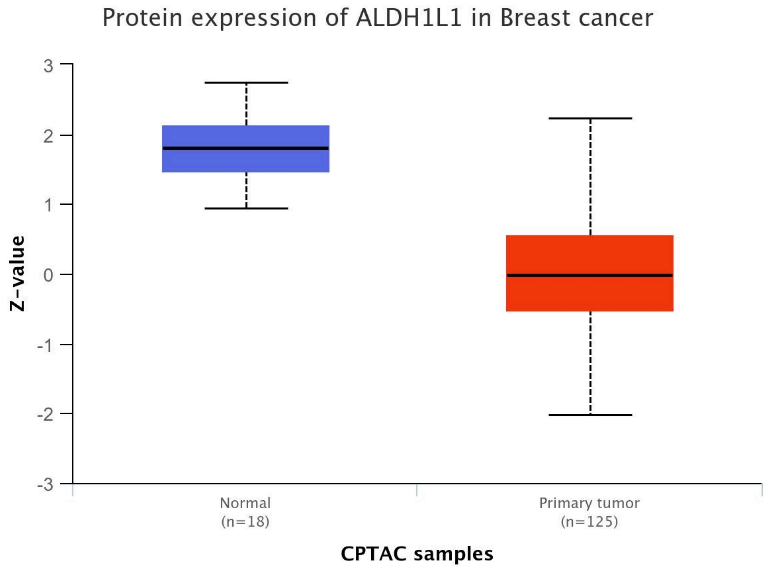

Supplementary Figure S1.
Expression profiles of ALDH1L1 in breast cancer and normal tissues. **(A)** Expression profiles of ALDH1L1 mRNA levels in breast cancer and normal tissues (analyzed using the OncoDB portal, https://oncodb.org). **(B)** Expression profiles of ALDH1L1 protein levels in breast cancer and normal tissues (analyzed using CPTAC proteomic data from GEPIA2 portal, http://gepia2.cancer-pku.cn/#index ; $p < 0.001$).

Supplementary Figure 2

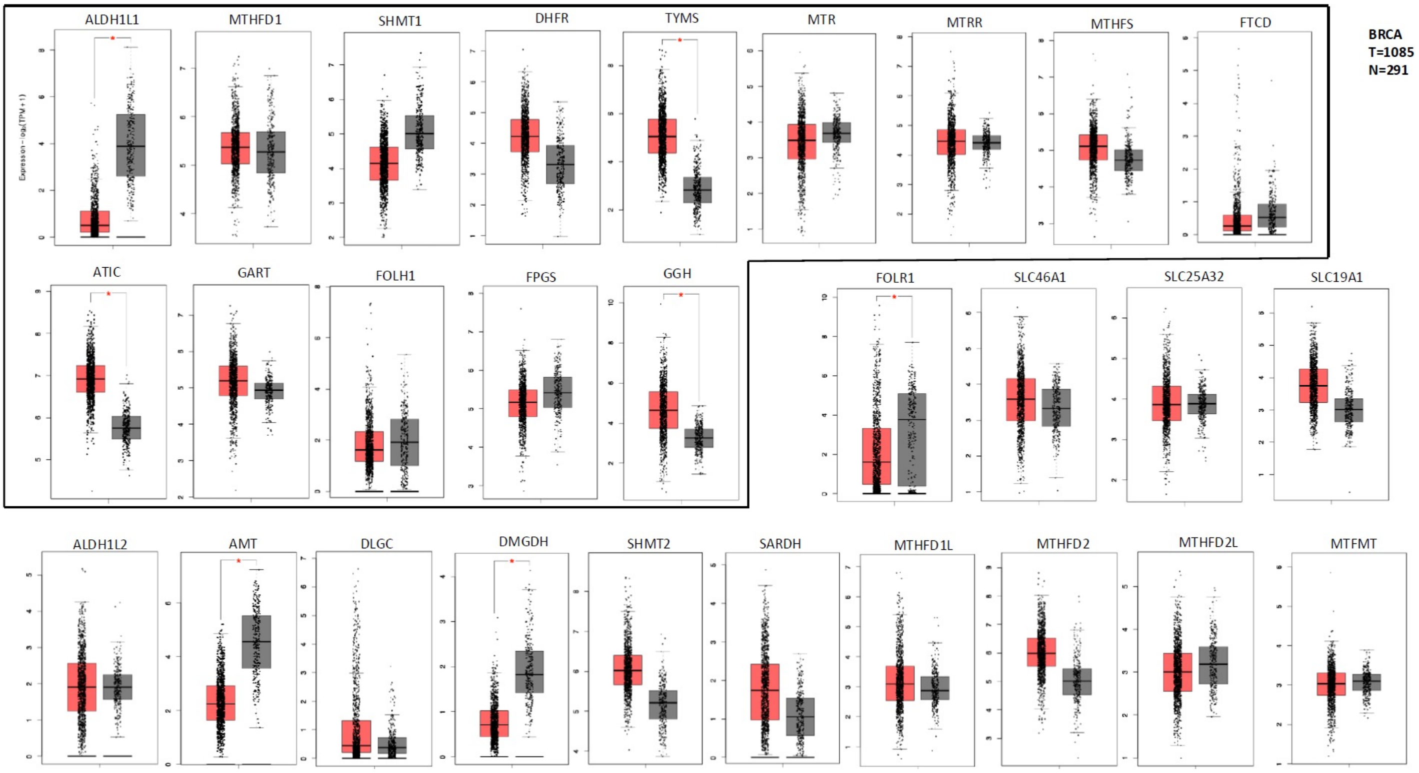

Supplementary Figure S2

Expression of mRNA of folate-metabolizing enzymes and transporters in breast cancer and normal tissues (analyzed using the GEPIA2, http://gepia2.cancer-pku.cn/#index). Red bars represent tumor tissues, and g ray bars represent normal tissues.

Supplementary Figure 3

A

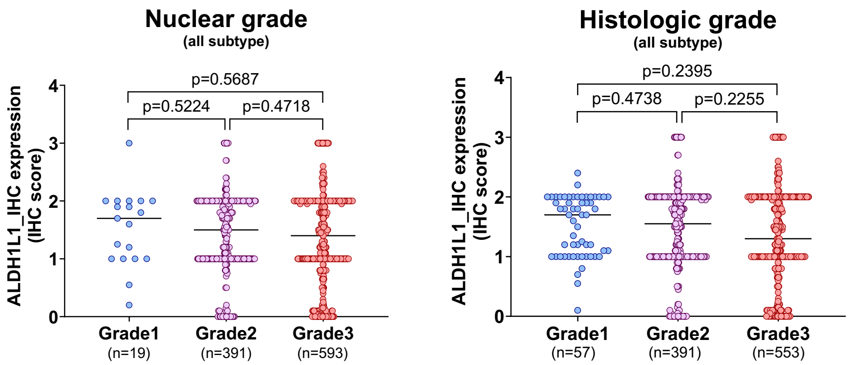

B

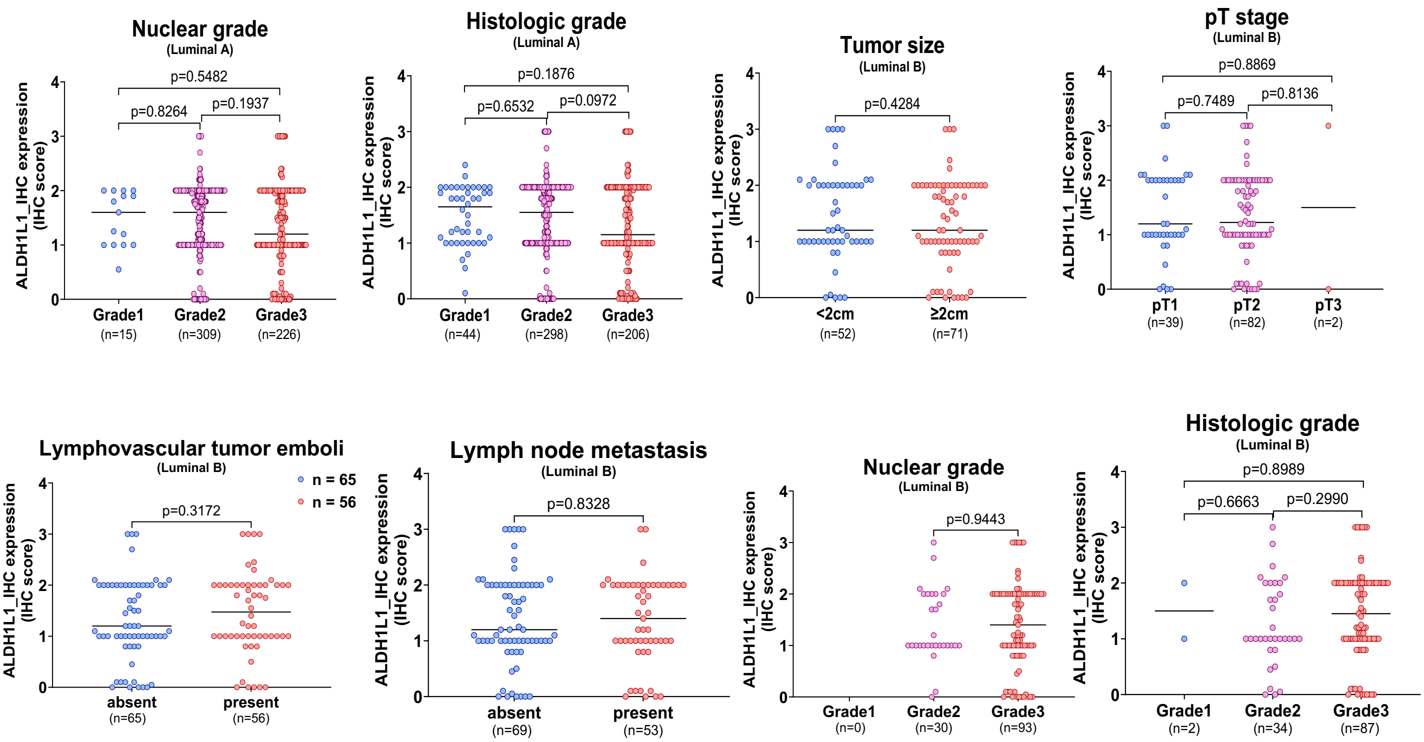

Supplementary Figure S3

Correlative analysis between clinicopathological parameters and ALDH1L1 expression level in breast cancer patients (statistically non-significant results). **(A)** ALDH1L1 protein expression and the variables in all breast cancer and **(B)** across breast cancer subtypes: Luminal A, Luminal B, HER2+, and TNBC from the SNUH cohort. ANOVA and the Student's t-test evaluated the association between ALDH1L1 and each clinicopathological variable.

Supplementary Figure 3

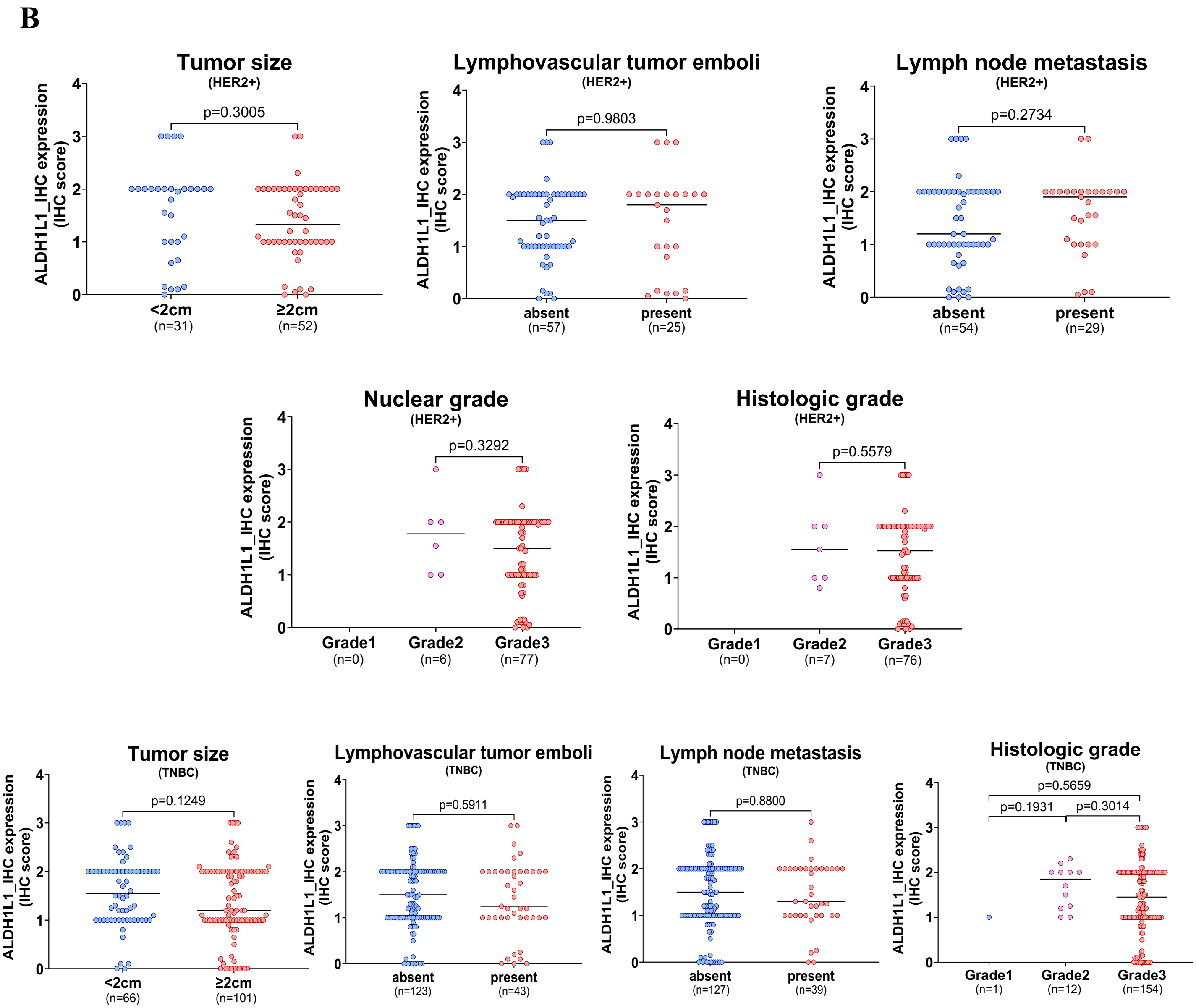

Supplementary Figure 4

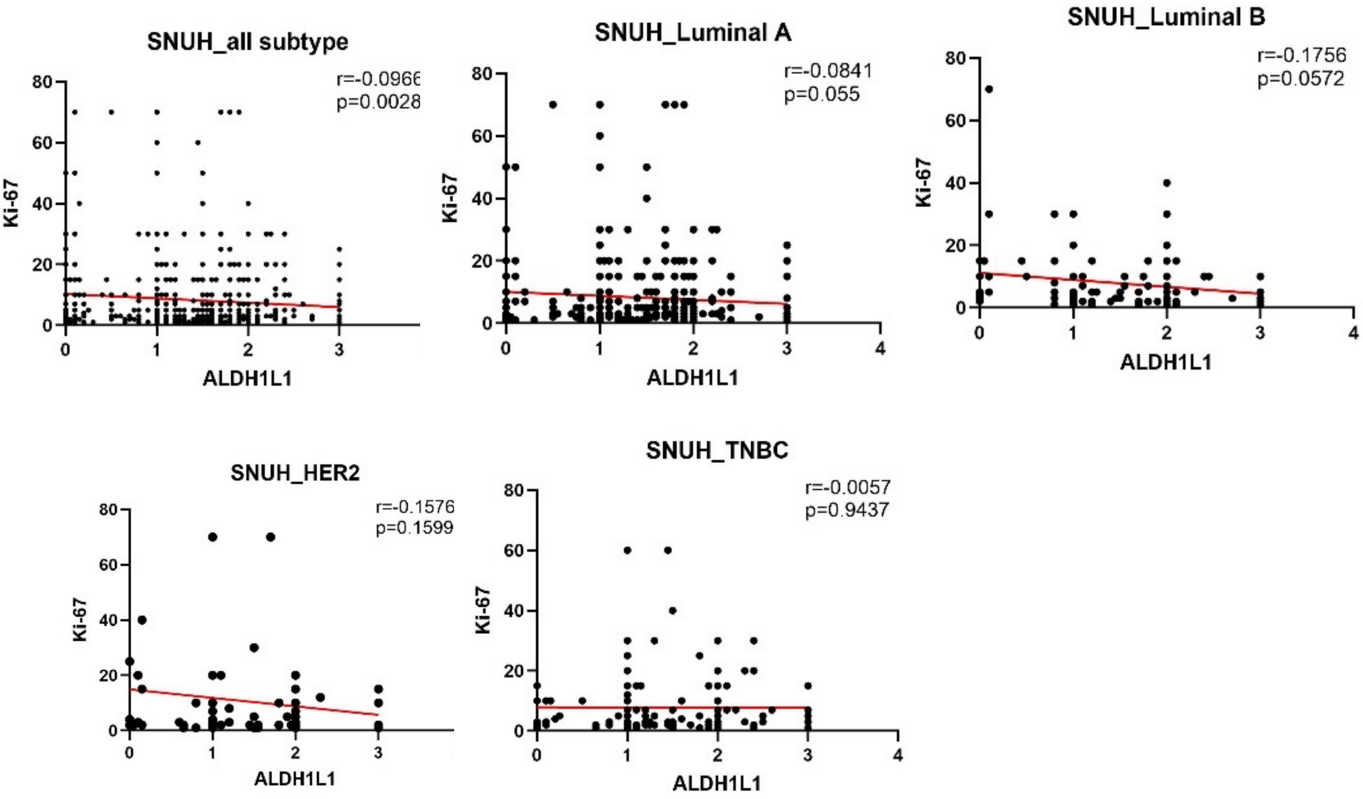

Supplementary Figure S4

Correlation analysis between ALDH1L1 expression, and Ki-67 in the SNUH tissue microarray (TMA) cohort comprising 1,001 invasive ductal carcinoma cases.

Supplementary Figure 5

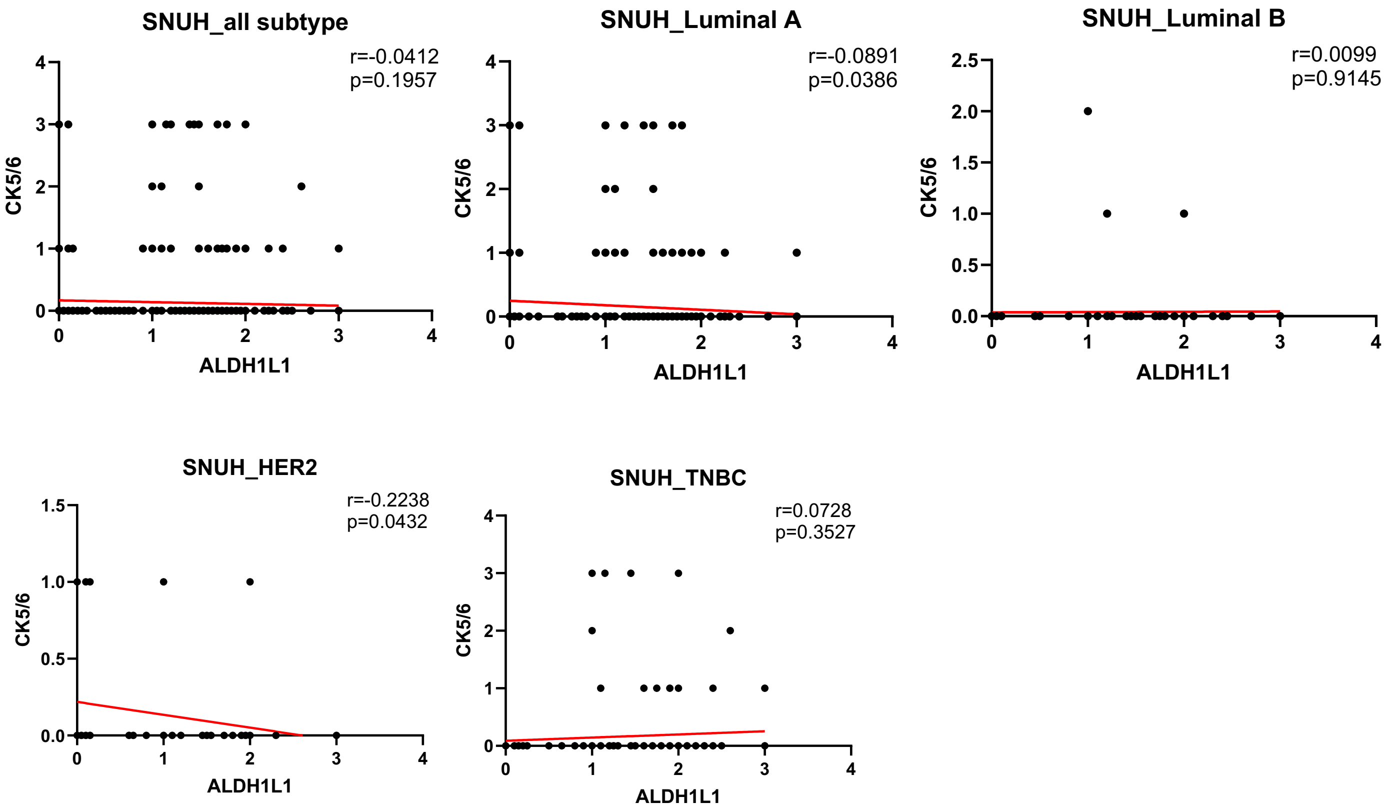

Supplementary Figure S5

Correlation analysis between ALDH1L1 expression, and CK5/6 in the SNUH tissue microarray (TMA) cohort comprising 1,001 invasive ductal carcinoma cases.

Supplementary Figure 6

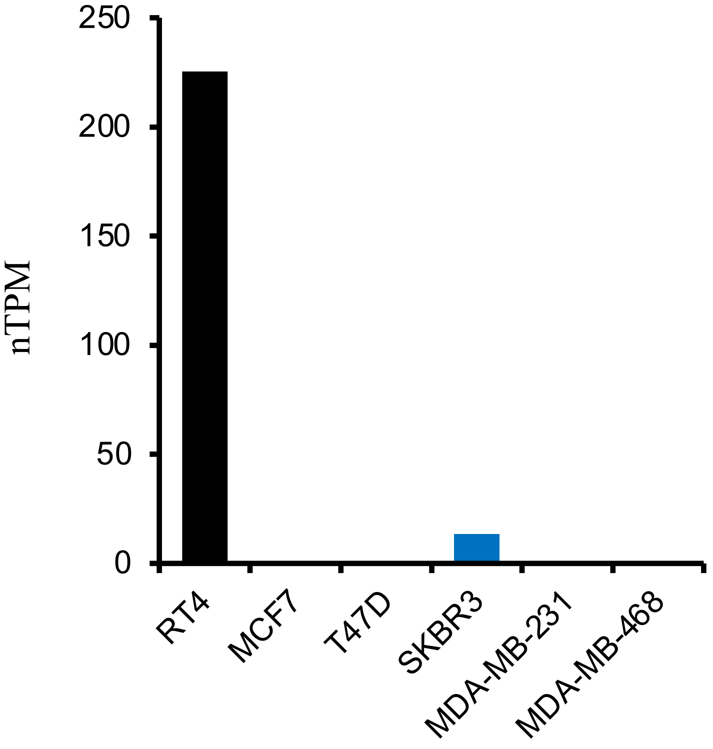

Supplementary Figure S6
Normalized mRNA expression of ALDH1L1 in breast cancer cell lines based on Human Protein Atlas (https://www.proteinatlas.org/)

Supplementary Figure 7

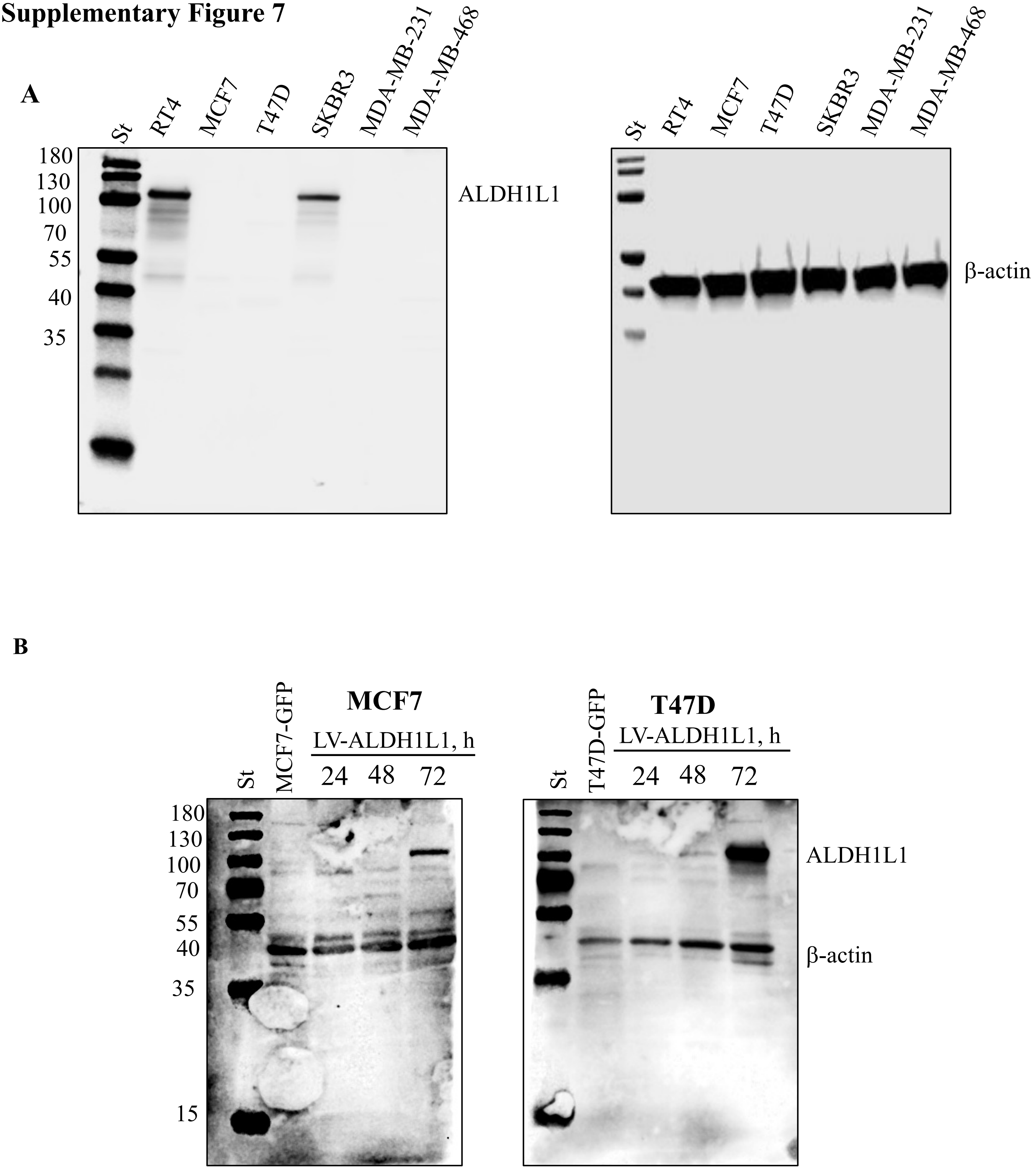

Supplementary Figure S7

(A) Original full-size blot corresponding to Figure 6A. **(B)** Original full-size blot corresponding to Figure 6B. NS, non-specific band. St, standard ladder.

Supplementary Figure 8

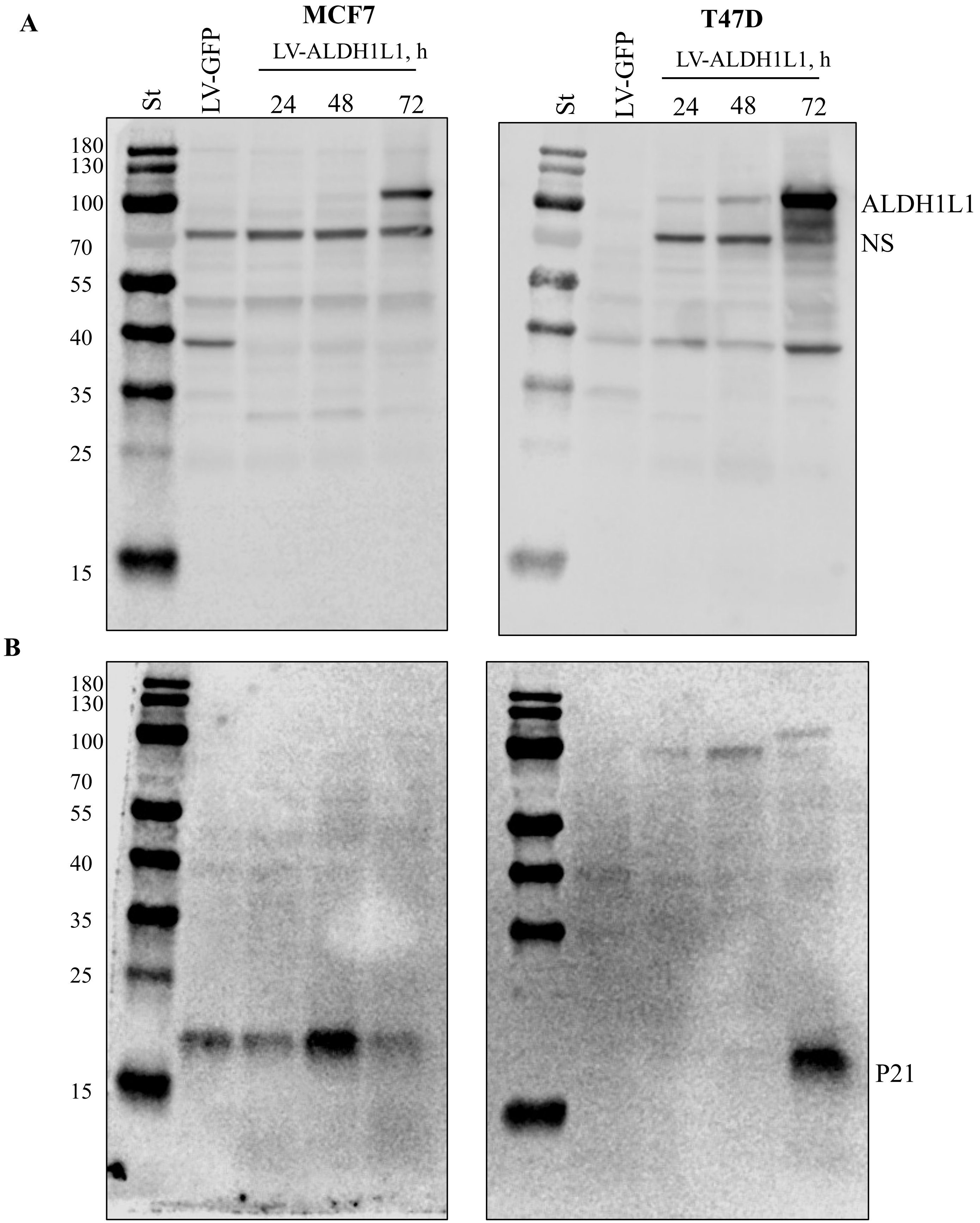

Supplementary Figure S8
(A-D) Original full-size blot corresponding to Figure 6E and 6H. NS, non-specific band. St, standard ladder.

Supplementary Figure 8

C

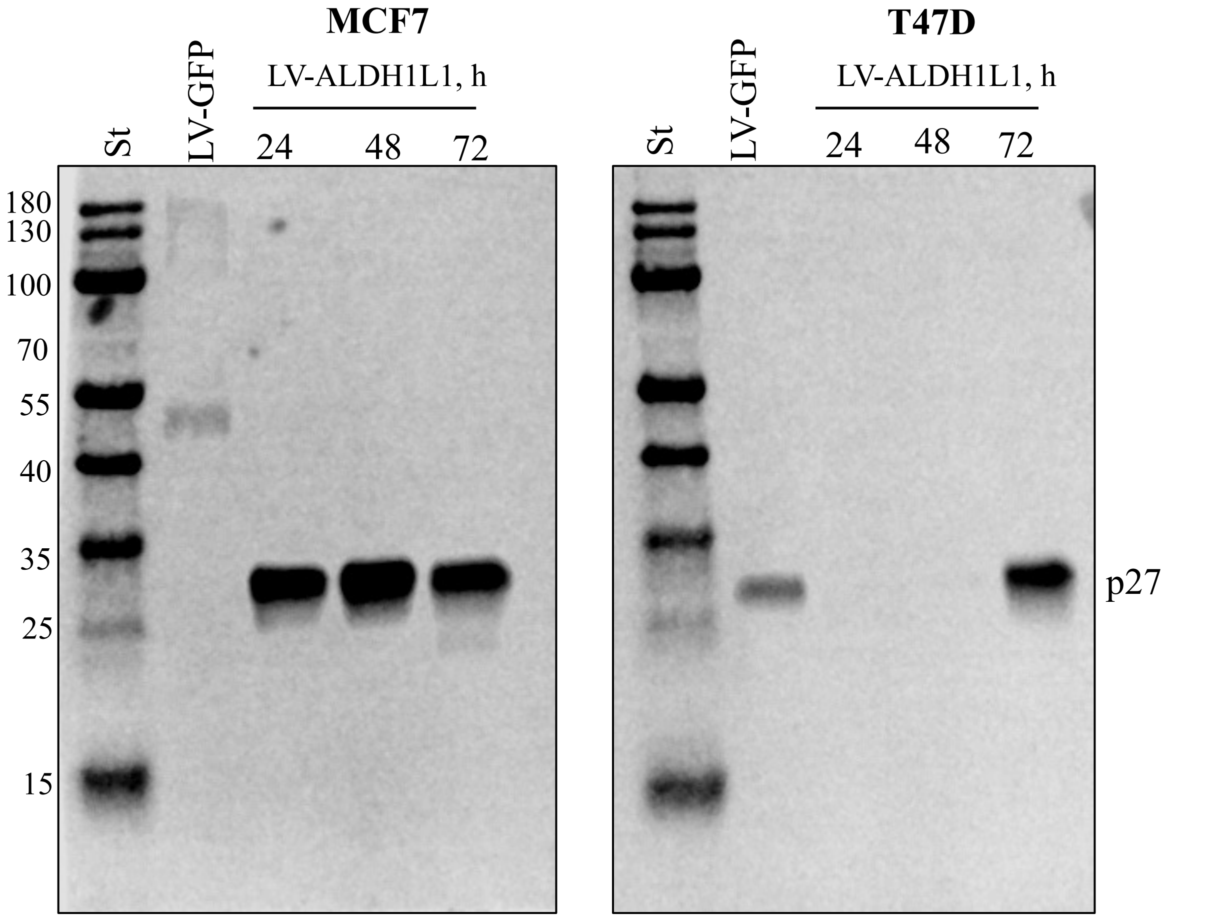

D

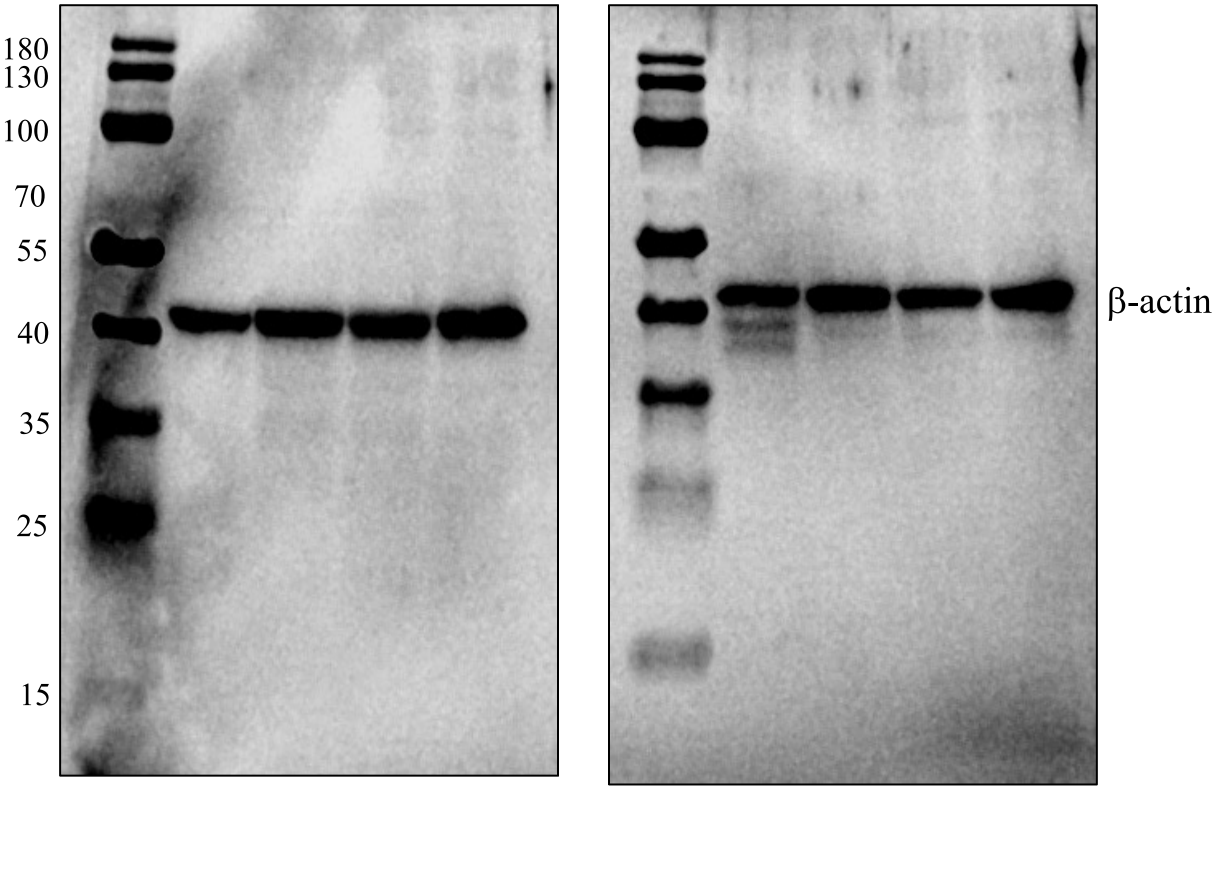

Supplementary Figure 9

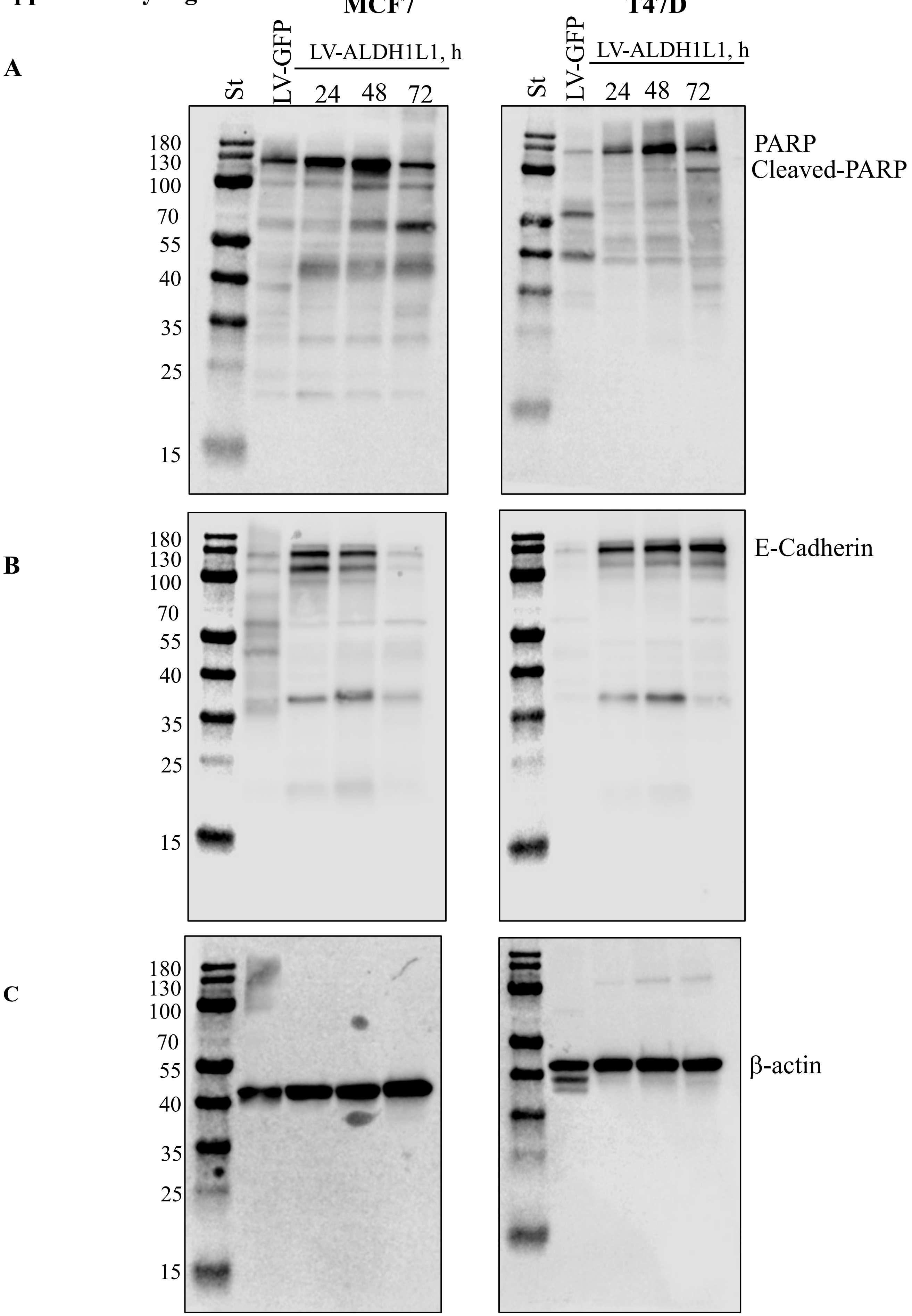

Supplementary Figure S9

(A-C) Original full-size blot corresponding to Figure 7A. St, standard ladder.
