# Supplementary material for: Clinical implications of 10-formyltetrahydrofolate dehydrogenase expression in hormone receptor-positive breast cancer
Source: Front Oncol. 2026 May 25;16:1838093. doi: 10.3389/fonc.2026.1838093 (PMC13243080; doi:10.3389/fonc.2026.1838093)
Supplement: Supplementary file 2 [file Table1.docx]

**Supplementary Table S1**

Spearman correlation coefficients (r) between ALDH1L1 expression and biomarkers of proliferation, metastasis, and recurrence across breast cancer subtypes (METABRIC cohort).

| Gene | All subtype  (n=1974) | Luminal A  (n=700) | | Luminal B  (n=475) | | HER2  (n=224) | | TNBC  (n=575) | |  |
| --- | --- | --- | --- | --- | --- | --- | --- | --- | --- | --- |
| CDH1 | -0.179 *** | -0.195 *** | | -0.144 ** | | -0.093 | | -0.184 *** | |  |
| CDH2 | -0.097 *** | -0.178 *** | | -0.042 | | -0.00004 | | -0.143 *** | |  |
| VIM | -0.265 *** | -0.307 *** | | -0.063 | | -0.113 | | -0.202 *** | |  |
| FN1 | -0.113 *** | -0.162 *** | | -0.040 | | -0.084 | | -0.101 * | |  |
| SNAI1 | -0.024 | -0.003 | | -0.020 | | -0.063 | | -0.103 * | |  |
| SNAI2 | -0.168 *** | -0.135 *** | | -0.004 | | -0.097 | | -0.122 ** | |  |
| MMP2 | -0.113 *** | -0.077 * | | -0.016 | | -0.040 | | -0.106 * | |  |
| MMP9 | -0.084 *** | -0.132 ** | | -0.076 | | -0.051 | | -0.192 *** | |  |
| MMP14 | -0.071 ** | -0.118 ** | | -0.071 | | -0.012 | | -0.074 | |  |
| CTSB | -0.041 | -0.060 | | -0.020 | | -0.020 | | -0.060 | |  |
| CTSD | -0.021 | -0.049 | | -0.021 | | -0.050 | | -0.043 | |  |
| CTSL | -0.060 ** | -0.096 * | | -0.019 | | -0.027 | | -0.010 | |  |
| VEGFA | -0.086 *** | -0.127 *** | | -0.026 | | -0.036 | | -0.160 *** | |  |
| CD44 | -0.006 | -0.062 | | -0.079 | | -0.053 | | -0.038 | |  |
| ALDH1A1 | -0.329 *** | -0.385 *** | | -0.093 * | | -0.082 | | -0.329 *** | |  |
| PROM1 | -0.093 *** | -0.119 ** | | -0.010 | | -0.096 | | -0.115 ** | |  |
| EPCAM | -0.122 *** | -0.007 | | -0.021 | | -0.185 ** | | -0.208 *** | |  |
| MKI67 | -0.166 *** | -0.113 ** | | -0.061 | | -0.138 * | | -0.259 *** | |  |
|  |  | |  | |  | |  | |  | |

Values are Spearman correlation coefficients (r).

Asterisks indicate statistical significance (*p<0.05, ** p <0.01, *** p <0.001).
