# Supplementary material for: Clinical implications of 10-formyltetrahydrofolate dehydrogenase expression in hormone receptor-positive breast cancer
Source: Front Oncol. 2026 May 25;16:1838093. doi: 10.3389/fonc.2026.1838093 (PMC13243080; doi:10.3389/fonc.2026.1838093)
Supplement: Supplementary file 3 [file Table2.docx]

**Supplementary Table S2** Correlation between ALDH1L1 expression and expression of oncogenic markers in breast cancer tumors (analyzed by GEPIA2).

| **No** | **Gene** | **Correlation value (R)** | **p-Value** | **No** | **Gene** | **Correlation value (R)** | **p-Value** |
| --- | --- | --- | --- | --- | --- | --- | --- |
| **1** | ACACB | 0.41 | 0 | **98** | ATIC | -0.13 | 0.000021 |
| **2** | CD36 | 0.38 | 0 | **99** | BRCA1 | -0.13 | 0.000021 |
| **3** | EBF1 | 0.35 | 0 | **100** | MYH11 | 0.13 | 1.5e−05 |
| **4** | PPARG | 0.33 | 0 | **101** | SPRED1 | 0.13 | 2.8e−05 |
| **5** | IGF1 | 0.32 | 0 | **102** | IL6 | 0.13 | 3.3e−05 |
| **6** | FOXO1 | 0.31 | 0 | **103** | TRIB3 | −0.13 | 1.8e−05 |
| **7** | ZBTB16 | 0.3 | 0 | **104** | RAD51C | −0.13 | 3.6e−05 |
| **8** | SGK1 | 0.28 | 0 | **105** | MOB3B | 0.13 | 2.5e−05 |
| **9** | DTX1 | 0.27 | 0 | **106** | ELOC | −0.13 | 1.4e−05 |
| **10** | TGFBR2 | 0.27 | 0 | **107** | ERRFI1 | 0.13 | 3.4e−05 |
| **11** | HLF | 0.26 | 0 | **108** | ESR1 | −0.13 | 3.1e−05 |
| **12** | KIT | 0.26 | 0 | **109** | ETV5 | 0.13 | 1.2e−05 |
| **13** | LIFR | 0.26 | 0 | **110** | BUB1B | −0.13 | 2.1e−05 |
| **14** | SOX17 | 0.26 | 0 | **111** | FGF1 | 0.13 | 1.2e−05 |
| **15** | TMPRSS2 | 0.26 | 0 | **112** | FGF2 | 0.13 | 1.7e−05 |
| **16** | EPAS1 | 0.25 | 0 | **113** | FLT1 | 0.13 | 2.9e−05 |
| **17** | PER1 | 0.24 | 2.2e−15 | **114** | GADD45B | 0.13 | 3.2e−05 |
| **18** | PREX2 | 0.24 | 1.8e−15 | **115** | HNRNPA2B1 | −0.13 | 1.2e−05 |
| **19** | PTPRB | 0.24 | 3.1e−15 | **116** | HOXA13 | 0.13 | 1.7e−05 |
| **20** | ADGRA2 | 0.24 | 3.6e−15 | **117** | JAZF1 | 0.13 | 1.3e−05 |
| **21** | ERG | 0.24 | 4.4e−16 | **118** | LMO2 | 0.13 | 1.9e−05 |
| **22** | ADGRG4 | 0.23 | 3.6e−14 | **119** | MAFB | 0.13 | 2.1e−05 |
| **23** | ARID5B | 0.22 | 6.8e−13 | **120** | BCL6 | 0.12 | 8.9e−05 |
| **24** | MAML2 | 0.22 | 6.4e−13 | **121** | IL7R | 0.12 | 0.00014 |
| **25** | TAL1 | 0.22 | 8.3e−14 | **122** | INSR | 0.12 | 5.4e−05 |
| **26** | TEK | 0.22 | 3e−13 | **123** | ID1 | 0.12 | 9.8e−05 |
| **27** | H6PD | 0.22 | 2.9E-13 | **124** | GATA3 | −0.12 | 7.7e−05 |
| **28** | KLF5 | 0.21 | 8.6e−13 | **125** | FOLR1 | 0.12 | 3.8e−05 |
| **29** | NOTCH4 | 0.21 | 2e−12 | **126** | LYL1 | 0.12 | 0.00011 |
| **30** | ROR1 | 0.21 | 7.1E-12 | **127** | MYB | −0.12 | 0.00014 |
| **31** | SLIT2 | 0.2 | 4.1e−11 | **128** | NR4A3 | 0.12 | 0.00011 |
| **32** | SLIT3 | 0.2 | 3.2e−11 | **129** | PNRC1 | 0.12 | 4.4e−05 |
| **33** | ETV1 | 0.2 | 1.2e−11 | **130** | PTCH1 | 0.12 | 0.00011 |
| **34** | GAS7 | 0.2 | 7.1e−11 | **131** | S1PR2 | 0.12 | 6.3e−05 |
| **35** | ALDH1A1 | 0.2 | 2.6E-11 | **132** | SESN1 | 0.12 | 0.00012 |
| **36** | CK4 | 0.2 | 4.5E-11 | **133** | SZT2 | 0.12 | 6.7e−05 |
| **37** | FAT4 | 0.19 | 2.4e−10 | **134** | VEGFB | 0.12 | 1e−04 |
| **38** | ALDH2 | 0.19 | 4.1e−10 | **135** | FZD7 | 0.12 | 0.000043 |
| **39** | FLI1 | 0.19 | 4.4e−10 | **136** | FBXW7 | 0.11 | 0.00028 |
| **40** | PAK3 | 0.19 | 1.7e−10 | **137** | FES | 0.11 | 0.00017 |
| **41** | RUNX1T1 | 0.19 | 1.9e−10 | **138** | FANCI | −0.11 | 0.00045 |
| **42** | CDKN1C | 0.18 | 2.5e−09 | **139** | FANCD2 | −0.11 | 0.00017 |
| **43** | DDR2 | 0.18 | 1.9e−09 | **140** | FOXO4 | 0.11 | 0.00023 |
| **44** | STAT5A | 0.18 | 1.3e−09 | **141** | HSP90AB1 | −0.11 | 4e−04 |
| **45** | SFRP1 | 0.18 | 1.8e−09 | **142** | KNSTRN | −0.11 | 0.00036 |
| **46** | TFEB | 0.18 | 5.5e−09 | **143** | MPL | 0.11 | 0.00041 |
| **47** | ZFP36L2 | 0.18 | 2.1e−09 | **144** | MTHFD2 | −0.11 | 0.00043 |
| **48** | ICAM2 | 0.18 | 1.8E-09 | **145** | NFATC2 | 0.11 | 4e−04 |
| **49** | FLT4 | 0.17 | 1.6e−08 | **146** | PARP1 | −0.11 | 0.00042 |
| **50** | SDHD | 0.17 | 1.8e−08 | **147** | PDGFRB | 0.11 | 2e−04 |
| **51** | SOCS3 | 0.17 | 2.3e−08 | **148** | POLQ | −0.11 | 2e−04 |
| **52** | TCF7L2 | 0.17 | 2.6e−08 | **149** | PRDM1 | 0.11 | 0.00028 |
| **53** | GLI1 | 0.17 | 1.8e−08 | **150** | RAD54L | −0.11 | 5e−04 |
| **54** | LRIG3 | 0.17 | 1.1e−08 | **151** | SF3B2 | −0.11 | 0.00048 |
| **55** | ABCB1 | 0.17 | 8.7e−09 | **152** | SOCS2 | 0.11 | 0.00046 |
| **56** | DUSP22 | 0.17 | 2.8e−08 | **153** | STAT5B | 0.11 | 0.00026 |
| **57** | ACVR2A | 0.16 | 8.3e−08 | **154** | TNFAIP3 | 0.11 | 0.00052 |
| **58** | CEBPA | 0.16 | 6.1e−08 | **155** | TNFRSF11A | 0.11 | 4e−04 |
| **59** | EGR1 | 0.16 | 6.7e−08 | **156** | TRIP13 | −0.11 | 0.00027 |
| **60** | EGR2 | 0.16 | 2.1e−07 | **157** | ZFP36L1 | 0.11 | 3e−04 |
| **61** | HSD17B2 | 0.16 | 1.3e−07 | **158** | ZNF521 | 0.11 | 0.00037 |
| **62** | TLE4 | 0.16 | 1.4e−07 | **159** | BCL11B | 0.11 | 0.00033 |
| **63** | KDR | 0.16 | 1.2e−07 | **160** | AURKA | −0.11 | 0.00037 |
| **64** | KLF2 | 0.16 | 6.9e−08 | **161** | AURKB | −0.11 | 0.00039 |
| **65** | KLF4 | 0.16 | 1e−07 | **162** | ADHFE1 | 0.11 | 0.00045 |
| **66** | KLF6 | 0.16 | 5e−08 | **163** | CDKN2B | 0.11 | 0.00027 |
| **67** | LATS2 | 0.16 | 5.5e−08 | **164** | CHD4 | −0.11 | 0.00035 |
| **68** | MECOM | 0.16 | 2.6e−07 | **165** | ESCO2 | −0.11 | 0.00053 |
| **69** | PAK7 | 0.16 | 7.1e−08 | **166** | BIRC5 | -0.11 | 0.00043 |
| **70** | ESR2 | 0.16 | 0.00000014 | **167** | PRKAG1 | -0.11 | 0.0004 |
| **71** | MET | 0.15 | 7.9e−07 | **168** | TMED2 | -0.11 | 0.00031 |
| **72** | NIN | 0.15 | 4.9e−07 | **169** | DPYD | 0.1 | 6e−04 |
| **73** | PTPN14 | 0.15 | 1.3e−06 | **170** | EPHB1 | 0.1 | 0.00059 |
| **74** | RAD51 | −0.15 | 1e−06 | **171** | CDH1 | 0.1 | 0.00057 |
| **75** | TP63 | 0.15 | 1.1e−06 | **172** | BSAC | 0.1 | 0.001 |
| **76** | TYRO3 | 0.15 | 3.2e−07 | **173** | ATP1A1 | 0.1 | 0.001 |
| **78** | ETS1 | 0.15 | 8e−07 | **174** | ARHGEF28 | 0.1 | 0.00083 |
| **79** | INPP5D | 0.15 | 9.3e−07 | **175** | ACVR1 | 0.1 | 0.00076 |
| **80** | IFNGR1 | 0.15 | 5.3e−07 | **176** | EZR | −0.1 | 0.00063 |
| **81** | BACH2 | 0.15 | 5.3e−07 | **177** | FANCF | −0.1 | 7e−04 |
| **82** | FGF7 | 0.15 | 1.5e−06 | **178** | FANCG | −0.1 | 0.00061 |
| **83** | FSTL1 | 0.15 | 4.5e−07 | **179** | ID3 | 0.1 | 0.00085 |
| **84** | TPT1 | 0.15 | 0.00000037 | **180** | MERTK | 0.1 | 0.00059 |
| **85** | APLNR | 0.14 | 4.9e−06 | **181** | PCSK7 | 0.1 | 0.00055 |
| **86** | ELN | 0.14 | 2.2e−06 | **182** | PDGFRA | 0.1 | 8e−04 |
| **87** | JUN | 0.14 | 3.4e−06 | **183** | PIM1 | 0.1 | 0.00089 |
| **88** | MAF | 0.14 | 3.9e−06 | **184** | PRDM16 | 0.1 | 0.00088 |
| **89** | MEF2C | 0.14 | 2.7e−06 | **185** | SOX10 | 0.1 | 0.00057 |
| **90** | NOD1 | 0.14 | 2.1e−06 | **186** | TONSL | −0.1 | 0.00075 |
| **91** | NONO | −0.14 | 5.4e−06 | **187** | TOP2A | −0.1 | 0.00079 |
| **92** | SFRP2 | 0.14 | 4.5e−06 | **188** | YAP1 | 0.1 | 0.00057 |
| **93** | SFRP4 | 0.14 | 2.3e−06 | **189** | ZBTB20 | 0.1 | 0.00058 |
| **94** | CCNB1 | -0.14 | 0.000045 | **190** | GPR177 | 0.1 | 0.00075 |
| **95** | PRKAA1 | 0.14 | 0.0000069 | **191** | CFL1 | -0.1 | 0.00064 |
| **96** | PAI1 | 0.13 | 0.000027 | **192** | CFL2 | 0.1 | 0.00018 |
| **97** | UBE2C | -0.13 | 0.000031 | **193** | MKI67 (Ki-67) | -0.089 | 0.0033 |

Pearson correlation coefficient**(PCC)** analysis has been used to measure the correlation between two genes using TCGA data.
