# Supplementary material for: Clinical implications of 10-formyltetrahydrofolate dehydrogenase expression in hormone receptor-positive breast cancer
Source: Front Oncol. 2026 May 25;16:1838093. doi: 10.3389/fonc.2026.1838093 (PMC13243080; doi:10.3389/fonc.2026.1838093)
Supplement: Supplementary file 4 [file Table3.docx]

**Supplementary Table S3** Luminal breast cancer cell lines analyzed for mRNA levels of folate enzymes.

| **No.** | **Luminal breast cancer cells** |
| --- | --- |
| 1 | T-47D |
| 2 | ZR-75-30 |
| 3 | MDA-MB-361 |
| 4 | ZR-75-1 |
| 5 | CAMA-1 |
| 6 | MCF-7 |
| 7 | MDA-MB-330 |
| 8 | BT-474 |
| 9 | UACC-812 |
| 10 | MDA-MB-175-VII |
| 11 | UACC-893 |
| 12 | EFM-19 |
| 13 | MDA-MB-415 |
